# Structural basis for domain coupling in heteromeric glycine receptors revealed by an atypical allosteric agonist

**DOI:** 10.1126/sciadv.aeb2036

**Published:** 2026-02-13

**Authors:** Eric Gibbs, Bjarne Feddersen, Kayla J. Kindig, David Seiferth, Philip C. Biggin, Sudha Chakrapani

**Affiliations:** ^1^ Department of Pharmacology, Case Western Reserve University, Cleveland, OH 44106-4970, USA.; ^2^ Structural Bioinformatics and Computational Biochemistry, Department of Biochemistry, University of Oxford, Oxford OX1 3QU, UK.; ^3^ Cleveland Center for Membrane and Structural Biology, Case Western Reserve University, Cleveland, OH 44106-4970, USA.

## Abstract

Glycine receptors (GlyRs), pentameric ligand-gated ion channels (pLGICs), mediate sensory and motor functions. GlyR functional states are well characterized; however, structural details of transitions between states remain undefined. Here, we determined cryo–electron microscopy structures of GlyRα1β (with gephyrin E-domain) at varying concentrations of ivermectin, a transmembrane domain (TMD) allosteric agonist, and at saturating concentrations of strychnine, a competitive antagonist at the extracellular domain (ECD). Electrophysiology shows that ivermectin activates GlyR even with strychnine present. Structures with both ligands reveal intermediate states featuring a desensitized TMD and an ECD between closed and desensitized conformations, providing insights into domain cooperativity and ligand efficacy. Molecular dynamics simulations show how ivermectin affects strychnine dynamics. These data support a model where ivermectin activates GlyRs through a concerted and near-symmetric TMD mechanism, whereas allosteric ECD motions are graded and spatially heterogeneous. These findings reveal unanticipated features of GlyR gating and establish principles of allosteric modulation applicable to pLGICs.

## Teaser

Counteracting ligands stabilize glycine receptor states revealing mechanisms of pentameric ligand-gated channel allostery.

## INTRODUCTION

Pentameric ligand-gated ion channels (pLGICs) in vertebrates include prominent synaptic receptors such as nicotinic acetylcholine receptors (nAChRs), 5-hydroxytryptamine type 3 receptors (5-HT_3_Rs), γ-aminobutyric acid type A receptors (GABA_A_Rs), and glycine receptors (GlyRs). These channels are critical for synaptic communication, and their dysfunction underlies many pathological disorders (*1*). As such, there is a long-standing interest in pLGIC structure and function both for their fundamental importance and therapeutic potential. This work focuses on heteromeric GlyR, a pLGIC that provides inhibitory input in the spinal cord for several processes, most notably motor control (*1*). Major disruption of GlyR signaling is lethal, and minor disruptions, such as those caused by genetic mutation, lead to movement disorders like hyperekplexia (*2*). Heteromeric GlyR is composed of two types of subunits, α and β. There are four mammalian α subunits (α1 to α4), with differential expression patterns across the body and in different species, α1 being the most widely expressed (*1*). Synaptic GlyR is necessarily heteromeric because α subunits are required for channel function and β subunits are necessary to bind gephyrin, an interaction essential for synaptic localization (*3*). Gephyrin is an intracellular protein that organizes GlyR and other integral and peripheral membrane proteins. Deleterious mutations in gephyrin also induce hyperekplexia, demonstrating its critical role in GlyR signaling (*3*). Gephyrin has three domains: the G domain, which self-trimerizes; the E domain, which self-dimerizes and binds the β subunit of GlyR; and the flexible C domain, which forms an extended linker between E and G domains (*4*). With multiple oligomerization states, gephyrin forms an extended lattice that anchors many heteromeric GlyRs as part of the inhibitory synaptic space (*4*).

Rapid synaptic communication requires that ion channels transition from an unliganded resting state to liganded conformations including transient pre-open and open states, followed by a stable desensitized state. Additionally, kinetic modeling of pLGIC electrophysiology data shows that there are multiple states within these broad categories (*5*, *6*). A simplistic approach used in kinetic modeling is to assume a single activation pathway that connects the closed, open, and desensitized states. Ligand binding shifts the free energy landscape and, hence, the relative equilibrium population of each state (*5*, *6*). While the kinetics between gating states are well established through single-channel analysis, the structural details governing these transitions are just beginning to emerge (*7*–*9*). These are important as many physiological, clinical, and recreational compounds act as allosteric modulators of pLGICs (*10*, *11*).

Decades of structural findings have provided the framework for connecting kinetic models to physical systems (*1*, *12*). These studies showed that five pLGIC subunits are arranged pseudosymmetrically about the channel pore axis and the receptor can be divided into extracellular, transmembrane, and intracellular domains (ECD, TMD, and ICD, respectively). Each subunit’s ECD is composed of 10 β strands (β1 to β10) and connecting loops. The TMD is composed of four α helices (M1 to M4), of which M2 forms the channel pore. Most pLGICs also have an extended intracellular loop between M3 and M4 that forms the ICD. Orthosteric ligands bind at subunit interfaces within the ECD, about 70 Å distal to the membrane. Allosteric coupling between the ECD and TMD leads to transitions in the channel pore through the closed, open, and desensitized states and is thus critical to receptor function (*13*).

Chimeric pLGICs and cryo–electron microscopy (cryo-EM) studies have identified structural elements important to domain coupling (*14*–*18*). The ECD coupling regions include the β1-β2 loop, β6-β7 loop (Cys-loop) at the primary interface, and the β8-β9 loop and the pre-M1 linker region at the complementary interface. These surround the M2-M3 loop, which protrudes up from the membrane interface. The allosteric arrangements that accompany ligand binding cause a rotation of the ECD, which pulls the M2-M3 loop further from the membrane. This leads to an expansion at the extracellular end of the M2 helix that accompanies channel activation and eventual desensitization. Often, channel activation is described as a unidirectional “conformational wave” from the orthosteric binding site to the pore (*6*, *19*). A key feature of allosteric modulation is that domain coupling is reciprocal. Hence, modulators that bind within the TMD or ICD will also affect orthosteric ligand efficacy, as is seen for various lipidic allosteric modulators or posttranslational modifications (*20*–*22*). The general mechanism of ECD/TMD coupling is conserved across pLGICs such that chimeric channels that have the ECD from one subtype and TMD of another retain some degree of channel activity (*14*–*16*). These studies show that there is cooperativity between subunits and that channel activity occurs only after the full transition of each subunit. Despite similarities across the family, recent studies show that the energetic cost of rearranging the M2-M3 loop can differ between subunits in heteromeric channels including GABA_A_Rs and nAChRs (*7*, *23*). Furthermore, asymmetric transitions that precede opening in nAChR and GLIC have recently been explored (*9*, *24*).

To better understand ECD/TMD coupling in heteromeric GlyR, we studied the structure and function of GlyR under the effects of opposing ligands, strychnine and ivermectin. Strychnine is an orthosteric antagonist of GlyR known for its acute, nanomolar toxicity (*1*). Past structural studies have shown that it acts by stabilizing the closed state of GlyR. Ivermectin, on the other hand, binds to an allosteric site within the TMD at subunit interfaces near the extracellular membrane leaflet. This site lies directly below the M2-M3 loop and is positioned to affect coupling between the ECD and TMD (*11*, *18*, *25*). Other allosteric modulators, such as *n*-alcohols and anesthetics, share this site, highlighting its physiological relevance. Ivermectin acts as a positive allosteric modulator (PAM) of glycine currents at low micromolar concentrations and as an agonist at higher concentrations (*26*). Ivermectin currents are distinct from glycine currents as they are insensitive to strychnine and picrotoxin and slow to desensitize (*26*, *27*).

The unusual properties of ivermectin led us to three points of inquiry related to the mechanisms of allosteric regulation of GlyR. First, does ivermectin activation occur by stabilizing the same states as orthosteric agonists or in another manner entirely? Past structures of GlyRs in the presence of both ivermectin and glycine are in the desensitized conformation resembling the states with glycine alone (*17*, *28*, *29*). However, it may be that, while ivermectin potentiation of glycine currents occurs along this pathway, activation by ivermectin alone occurs in a different manner. We hypothesized that studying ivermectin in the presence of strychnine would isolate features of ivermectin activation distinct from those of glycine. The second question was whether the opposing action of strychnine and ivermectin could stabilize intermediate states that are not otherwise observed. There is interest in capturing intermediate states of pLGICs through either releasing photocaged ligands, passing grids through a nanojet spray of ligand during plunge freezing, or using slow-acting ligands (*8*, *30*, *31*). Our approach is not time dependent and is designed to capture equilibrium states. However, the extent of activation may differ among these states and reveal mechanisms of channel allostery. Last, we are interested in the ligand dynamics at the distinct heteromeric interfaces. There is increasing evidence that heteromeric and even homomeric receptor subunits act asymmetrically before channel opening, but the degree of asymmetry varies between subtypes (*9*, *23*, *32*, *33*). Understanding this asymmetry is important to therapeutic design as many existing pLGIC agonists or potentiators act at specific ligand interfaces. Heteromeric GlyR is an interesting case in that it only incorporates one asymmetric subunit, and some functionally important regions, such as the orthosteric binding site, are well conserved. This gives it properties somewhere between a homomeric pLGIC and more heterogeneous pLGICs such as most nAChRs and GABA_A_Rs. More compositionally distinct heteromers may have asymmetric mechanisms that arise from inherent differences, but we sought to understand the role of asymmetry in channels that are compositionally similar or identical such as heteromeric GlyR and homomeric pLGICs (*32*–*34*).

To accomplish these aims, we first verified and then further explored the ability of ivermectin to activate zebrafish (ZF) GlyRα1β in the presence of strychnine (*16*). We then collected multiple cryo-EM structures of ZF GlyRα1β with different concentrations of ivermectin (0.2, 0.5, 2, and 20 μM) with a constant saturating amount of strychnine (200 μM). At the higher two ivermectin concentrations, ivermectin and strychnine can simultaneously bind the channel. Molecular dynamics (MD) simulation was then used to better understand how strychnine binding is affected by ivermectin. The results provide details of allosteric coupling for GlyR and contribute to the general framework of allosteric coupling across pLGICs.

## RESULTS

### Functional validation and cryo-EM structure determination of heteromeric GlyR with ivermectin and strychnine

**Fig. 1.**
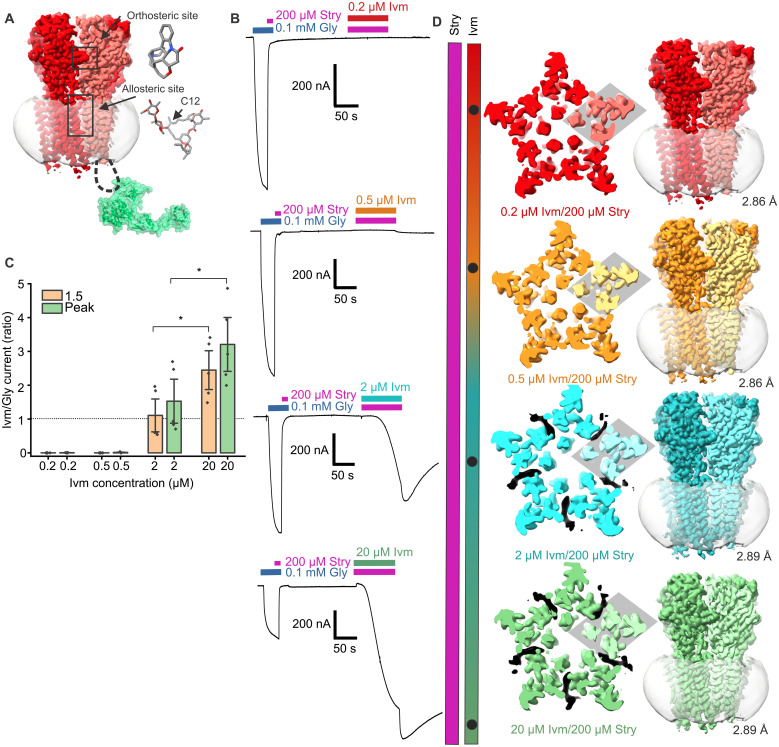
Electrophysiology data and summary of cryo-EM experiments. (**A**) Visual representation of the orthosteric (strychnine) and allosteric (ivermectin) binding sites referred to in this study. Domain E of gephyrin (E-Geph; Protein Data Bank: 2FU3) is shown in green, although it is not observed by cryo-EM. (**B**) Electrophysiology data demonstrating the effects of coapplication of ivermectin and strychnine. Representative TEVC traces in which a subsaturating pulse of glycine is applied and blocked by strychnine, followed by washout with continuous application of ND96, and then coapplication of 200 μM strychnine and ivermectin at varying concentrations. The ivermectin doses correspond to the cryo-EM experiments and are colored to match the cryo-EM maps. (**C**) Graph of the ratio of ivermectin currents to the maximum current at 100 μM glycine (*I*_Gly_), shown for the point at which ivermectin and strychnine application was shut off (1.5 min) and at *I*_max_ (after strychnine wash off). Data are presented as means ± SEM. At 1.5 min, the mean current ratio was 0.0003 ± 0.0003 for 0.2 μM (*n* = 5), 0.0004 ± 0.0004 for 0.5 μM (*n* = 5), 1.1 ± 0.3 for 2 μM (*n* = 5), and 2.4 ± 0.4 for 20 μM ivermectin (*n* = 5). At *I*_max_, the mean current ratio was 0.004 ± 0.002 for 0.2 μM (*n* = 5), 0.01 ± 0.006 for 0.5 μM (*n* = 5), 1.5 ± 0.4 for 2 μM (*n* = 5), and 3.2 ± 0.5 for 20 μM ivermectin (*n* = 5). Statistical significance determined by Student’s *t* test; *P = 0.028 for 1.5 min and *P = 0.04 for *I*_max_. (**D**) Representative images and ligand conditions of the ivermectin/strychnine cryo-EM maps reported in this study. The cutaway shows the top of the TMD region, and ivermectin density, when present, is shown in black and is weaker at heteromeric interfaces. The β subunit is a lighter shade and highlighted with a gray diamond.

Our first goal was to characterize ivermectin activation currents for our constructs in the presence of a saturating concentration of strychnine (Fig. 1). To do this, we performed two-electrode voltage-clamp (TEVC) experiments with *Xenopus laevis* oocytes injected with mRNA for ZF GlyRα1, GlyRβb, and domain E of gephyrin (E-Geph). The GlyR constructs have been used in past structural and functional studies, and E-Geph was included to more faithfully represent the complex used for cryo-EM (*17*, *28*, *29*, *35*). The experimental protocol was to first measure the current in response to 100 μM glycine and show antagonism by 200 μM strychnine. After washout, 200 μM strychnine was coapplied with a variable dose of ivermectin. There was little to no current at the lower ivermectin concentrations (0.2 and 0.5 μM). At higher concentrations (2 and 20 μM), ivermectin currents activated and desensitized over the course of minutes (Fig. 1A and fig. S1), as previously observed (*26*). Notably, ligand removal during washout led to an immediate increase in ivermectin currents before deactivation. We attribute this to water-soluble strychnine being more easily washed off than ivermectin and ivermectin having an increased efficacy without strychnine. Ivermectin currents also did not return to baseline for several minutes, consistent with past work that shows complete ivermectin washout is not feasible for electrophysiology experiments (*26*). Gephyrin is known to affect GlyR functional properties, but the inclusion of E-Geph did not markedly change the relationship between strychnine and ivermectin activation, although there are possibly nuanced effects that are not captured by TEVC.

For structural studies, ZF GlyRα1 and GlyRβb were coexpressed with the E-Geph using the Expi-Sf9 baculovirus expression system. In past structural studies, E-Geph was included as a means to distinguish between subunits in cryo-EM reconstructions, but its binding site within the ICD was not resolved (*28*). Nonetheless, coexpression of E-Geph with heteromeric GlyR seemed to improve protein stability, and it was included in this study as well. As described in the methods, dual-affinity purification was performed using nickel–nitrilotriacetic acid (Ni-NTA) to select for 8xHis-tagged GlyRα1 and then using Sepharose beads conjugated to anti–Rho-1D4 to select for Rho-1D4–tagged GlyRβb (*36*). This was followed by size exclusion chromatography resulting in a distinct peak at the expected elution volume that contained both GlyR subunits. Coexpression of the GlyR subunits with E-Geph resulted in a ~0.7-ml leftward shift of this peak compared to that of GlyR subunits alone (*28*). The GlyRα1, GlyRβb, and E-Geph complex is referred to as GlyRα1β going forward.

Cryo-EM samples were prepared in the presence of 200 μM strychnine and 0.2, 0.5, 2, or 20 μM ivermectin (Fig. 1C). Data collection and processing resulted in a single high-resolution reconstruction and model for each condition (GlyRα1β-*X*Ivm200Stry, with *X* being 0.2, 0.5, 2, or 20). Each of the conditions led to a reconstruction with a fully resolved ECD and TMD. This includes portions that vary between α and β subunits, such as glycans and N- and C-terminal extensions. This allows for confident subunit alignment and identification. The ICD was only resolved through the first few residues, consistent with past anionic pLGIC structures (*10*, *17*, *18*, *28*, *37*). The reported resolutions are 2.86 Å for GlyRα1β-0.2Ivm200Stry, 2.86 Å for GlyRα1β-0.5Ivm200Stry, 2.89 Å for GlyRα1β-2Ivm200Stry, and 2.89 Å for GlyRα1β-20Ivm200Stry (Fig. 1D and figs. S2 to S9). The final map for GlyRα1β-20Ivm200Stry achieved comparable resolution to the other maps, but it required more particles that showed high-resolution features in two-dimensional (2D) classification. This may reflect greater heterogeneity in this dataset, which is expected considering the competing influence of strychnine and ivermectin.

### Overall conformation of ivermectin/strychnine structures and characterization of the channel pore

Our structures demonstrated structural changes related to ivermectin occupancy in the presence of strychnine. The two lower ivermectin concentrations, GlyRα1β-0.2Ivm200Stry and GlyRα1β-0.5Ivm200Stry, have strychnine bound as expected, with no clear density for ivermectin, and the receptor conformation is generally consistent with past structures in a resting conformation (*17*, *28*, *29*, *37*). The two higher concentrations, GlyRα1β-2Ivm200Stry and GlyRα1β-20Ivm200Stry, have both strychnine and ivermectin bound at all five subunit interfaces, although the occupancy likely varies between the two states (Fig. 1D). These two states each have a distinct conformation that resembles a desensitized conformation within the TMD and an intermediate state between resting and desensitized states within the ECD, with GlyRα1β-20Ivm200Stry being more toward the desensitized state than GlyRα1β-2Ivm200Stry. To contextualize the intermediate positions of the strychnine/ivermectin structures, our analysis throughout the publication includes two previously reported GlyRα1β structures determined with the same experimental conditions except for ligand concentrations. A closed/inhibited state is represented by GlyRα1β in the presence of 100 μM strychnine (GlyRα1β-100Stry) and a desensitized state is represented by GlyRα1β in the presence of 20 μM ivermectin and 1 mM glycine (GlyRα1β-20Ivm1000Gly) (*28*). Other GlyR structural studies have also led to structures of closed and desensitized states that are generally comparable to these two structures, with some distinctions that may arise from the construct itself or the membrane mimetic environment (*17*, *28*, *29*, *33*, *37*, *38*).

**Fig. 2.**
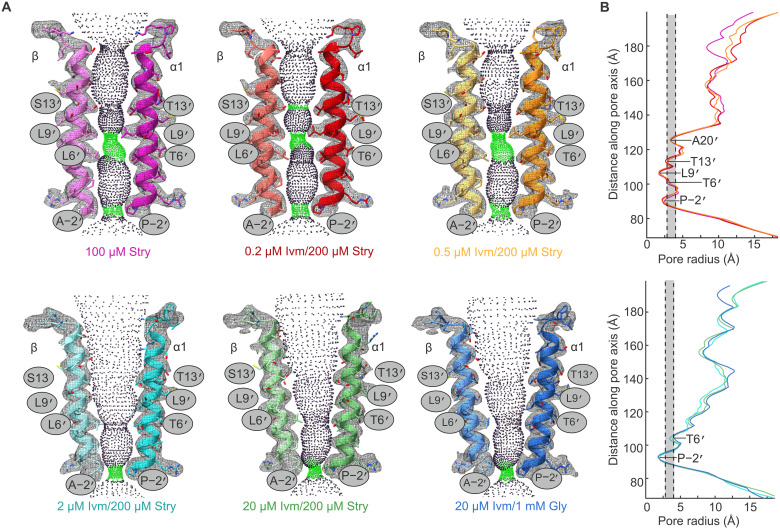
Analysis of the pore domain of ivermectin/strychnine structures. The pore of each reported structure is shown as well as two previously reported structures for heteromeric GlyR in the presence of saturating concentrations of strychnine alone (GlyRα1β-100Stry) and heteromeric GlyR in the presence of saturating ivermectin and glycine (GlyRα1β-20Ivm1000Gly). (**A**) Models of the M2 helix are shown with the cryo-EM data represented as a mesh and the calculated HOLE profile shown as colored dots. Green dots indicate where the pore radius can only accommodate one water molecule. The lower ivermectin structures clearly align well within the pore domain with the GlyRα1β-100Stry showing constrictions at both the Leu9′ and Pro/Ala−2′ positions. The higher ivermectin concentration structures have pores that are very similar to GlyRα1β-20Ivm1000Gly. (**B**) Plot showing the minimal pore radius from the extracellular (top) to intracellular end of the channel pore. Residues at narrow positions are labeled. The shaded region shows the region between the Born radius, first dashed line at 2.3 Å, and the radius of a fully hydrated chloride ion, second dashed line at 3.2 Å. These two values represent the range of pore radius values that could serve as a minimal radius to channel permeation.

The pore-lining M2 helices are nearly identical to previously characterized states. These helices of GlyRα1β-0.2Ivm200Stry and GlyRα1β-0.5Ivm200Stry align very closely with GlyRα1β-100Stry (Fig. 2 and fig. S10). Near the center of the pore, there is a substantial hydrophobic barrier formed by α1 Leu^285^ (β Leu^306^). A leucine in this position is conserved across the pLGIC family and is often referred to as Leu9′. The Leu9′ side chain faces toward the pore in the resting state and is rotated away from pore in open or desensitized states, forming the so-called “activation gate” (*18*). Using the program HOLE, the radius at this position is estimated to be 1.3, 1.4, and 1.3 Å for GlyRα1β-100Stry, GlyRα1β-0.2Ivm200Stry, and GlyRα1β-0.5Ivm200Stry, respectively (*39*). Another barrier to permeation is α1 Pro^274^ (β Ala^295^) at the intracellular end of M2. This Pro/Ala−2′ position is the most constricted point in the desensitized state and is referred to as the “desensitization gate” (*18*). Here, the pore radius was 1.8, 1.8, and 2.0 Å for GlyRα1β-100Stry, GlyRα1β-0.2Ivm200Stry, and GlyRα1β-0.5Ivm200Stry, respectively. Similarly, the C-α atoms at the Leu9′ and Pro/Ala−2′ positions show minimal changes in overall arrangement (fig. S10). While the pore radius at the desensitization gate is larger than the Pauling radius of a chloride ion (1.8 Å), the effects of solvent shielding and hydrophobicity also affect channel permeation (*40*). This makes it difficult to define a minimum radius threshold of permeable channels, but a reasonable range is the Born radius (2.3 Å) and the radius of a Cl^−^ ion with its hydration shell (3.2 Å) (*32*, *40*–*43*). In previous work, channel permeation was explored by MD simulation of GlyRα1β-100Stry, and both the activation and desensitization gate were permeation barriers in this state (*28*). Given their similarity to GlyRα1β-100Stry, we also assign GlyRα1β-0.2Ivm200Stry and GlyRα1β-0.5Ivm200Stry as closed conformations. This is consistent with the electrophysiology data that showed minimal currents at this ivermectin concentration. There are slight displacements for both GlyRα1β-0.2Ivm200Stry and GlyRα1β-0.5Ivm200Stry in the M1-M2 loop relative to GlyRα1β-100Stry. For both structures, the maximal C-α displacement in this region is 1.3 Å across all five subunits. These displacements are counterclockwise when viewed from the ECD, consistent with rearrangements toward the desensitized conformation. This suggests that GlyRα1β-0.2Ivm200Stry and GlyRα1β-0.5Ivm200Stry are slightly further along the activation pathway, although further conclusions are limited given the small changes.

Although this work only includes structures in the equilibrium desensitized states, past work has stabilized GlyR in the open state using pore blockers, different membrane conditions, or allosteric modulators (*10*, *29*, *37*, *38*). Channel activation leads to an outward displacement and a tilt of the TMD of each individual subunit. This rearranges the M2 helix so that the five helices are shaped like a funnel that is narrower at the cytosolic face. Leu9′ is rotated out, allowing for ion permeation. In the desensitized state, the M2 helices are arranged similarly but are slightly more compact at the cytosolic interface and form an ion conduction barrier at the Pro/Ala−2′ desensitization gate (*29*, *37*). For both GlyRα1β-2Ivm200Stry and GlyRα1β-20Ivm200Stry, the channel pore is like that of GlyRα1β-20Ivm1000Gly. The Leu9′ position is open, and the channel pore at the Pro/Ala−2′ position is constricted to 1.6, 1.4, and 1.4 Å for GlyRα1β-2Ivm200Stry, GlyRα1β-20Ivm200Stry, and GlyRα1β-20Ivm1000Gly, respectively (Fig. 2). The C-α positions at the Leu9′ and Pro/Ala−2′ positions are also very similar between these three structures (fig. S10). Like the strychnine-inhibited state, GlyRα1β-20Ivm1000Gly has been previously characterized by MD simulation, and, hence, we annotate GlyRα1β-2Ivm200Stry and GlyRα1β-20Ivm200Stry as desensitized states (*28*). Although the conformation at the Pro/Ala−2′ is similar among the ivermectin/strychnine structures, the M1-M2 loop is displaced, counterclockwise when viewed from the ECD, toward the closed conformation compared to GlyRα1β-20Ivm1000Gly. The maximal C-α displacement in this region is 1.8 Å in the GlyRα1β-2Ivm200Stry model relative to GlyRα1β-20Ivm1000Gly. This suggests that GlyRα1β-2Ivm200Stry and GlyRα1β-20Ivm200Stry may occupy states along the pathway connecting GlyRα1β-100Stry to GlyRα1β-20Ivm1000Gly.

Despite slight differences within each group, there is clear segregation between the closed and fully desensitized states. This abrupt shift occurs between 0.5 and 2 μM ivermectin, again consistent with TEVC data (Fig. 1). All the high-resolution structures have a channel pore that is mostly symmetric, which aligns with past functional data. For example, one study using chimeric pLGICs showed that pentamers that include subunits with defective ECD/TMD coupling have reduced mean open times but similar single-channel conductance values (*15*). Recent work shows that sequential binding within the orthosteric site can induce asynchronous changes within the pore, but accessing the open state requires a concerted transition of the five M2 helices (*9*, *24*). Together, these observations support that ivermectin application leads to a concerted and near-symmetric transition within the channel pore, suggesting a high degree of cooperativity in the TMD movement.

### Ivermectin binding site

**Fig. 3.**
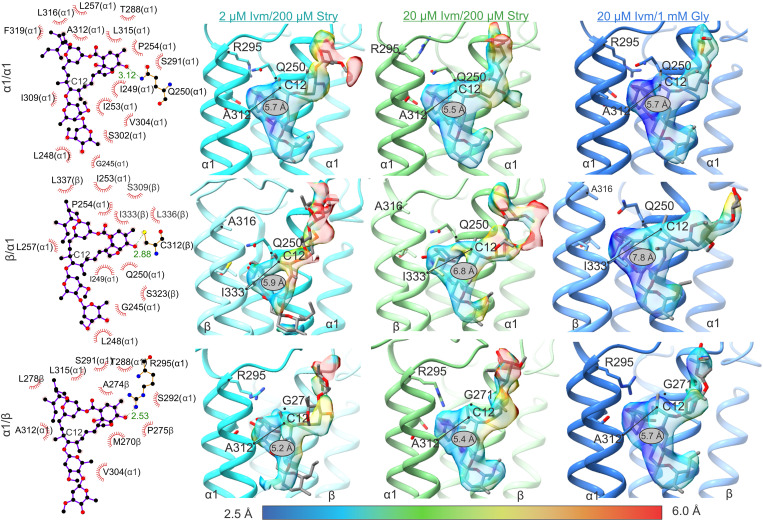
Ivermectin binding site. LigPlot analysis, models, and ivermectin density are shown for GlyRα1β-2Ivm200Stry and GlyRα1β-20Ivm200Stry at an α1/α1, β/α1, and α1/β interface. The ivermectin density observed in a previous study of GlyRα1β-20Ivm1000Gly is also included for comparison. LigPlot analysis, shown in left column, is shown at the three interface types from the GlyRα1β-2Ivm200Stry, but the results were not substantially different for GlyRα1β-20Ivm200Stry or GlyRα1β-20Ivm1000Gly. The ivermectin density is colored on the basis of the local resolution as determined by ResMap. The label shows the distance between the C-α atom of α1 Ala^312^ (β Ile^333^) and the C12 atom within the ivermectin lactone ring (C12 is labeled in Fig. 1). The ivermectin at the β/α1 interface is pushed toward the complementary interface due to the bulky side chain of β Ile^333^, although the density is too poor to definitively observe this in GlyRα1β-2Ivm200Stry. Both the resolution and continuity of the ivermectin density is less at the α1/β and β/α1 interface, especially for GlyRα1β-2Ivm200Stry. This is likely due to reduced occupancy caused by cross-talk with the orthosteric binding site.

The ivermectin binding site has been previously described to be in the extracellular membrane leaflet between the M3 helix on the primary subunit (clockwise when viewed from the extracellular space) and M1 helix on the complementary subunit (counterclockwise when viewed from the extracellular space) (*17*, *18*, *28*, *29*). The interactions are mostly between the lactone core of ivermectin and multiple hydrophobic residues on the primary and complementary interface (Fig. 3 and fig. S11). Ivermectin density was observed at this site in all five interfaces of the desensitized state structures, GlyRα1β-2Ivm200Stry and GlyRα1β-20Ivm200Stry, but not at any interface in the closed state structures, GlyRα1β-0.2Ivm200Stry and GlyRα1β-0.5Ivm200Stry. Using the Fpocket algorithm, the ivermectin binding pocket has about 200 Å^3^ of accessible pocket volume for GlyRα1β-0.2Ivm200Stry and GlyRα1β-0.5Ivm200Stry (*44*). By contrast, the same interface in the GlyRα1β-2Ivm200Stry and GlyRα1β-20Ivm200Stry structures has about 700 Å^3^ of accessible pocket volume, suggesting that ivermectin binding at this site likely requires channel activation.

Although ivermectin density was observed at all interfaces of GlyRα1β-2Ivm200Stry and GlyRα1β-20Ivm200Stry, the density was reduced at the α1/β and β/α1 interface, especially in the GlyRα1β-2Ivm200Stry map. Several ligand/protein interactions at each heteromeric interface may contribute to weaker ivermectin binding at these interfaces (Fig. 3). Although hydrophobic interactions are the main drivers of ivermectin binding, the most notable differences at the α1/β interface involve polar side-chain interactions with ivermectin mediated by α1 Gln^250^ (β Gly^271^), located on M1, and α1 Arg^295^ (β Ala^316^), located on M2. At α1/α1 interfaces, α1 Gln^250^ interacts with the lactone oxygen, but this interaction is missing at the α1/β interface as β Gly^271^ has no side chain. In its place, α1 Arg^295^ from the primary interface extends outward from the pore and interacts with the ivermectin lactone oxygen. α1 Gln^250^ and Arg^295^ have been previously studied with regard to glycine and ivermectin channel activation (*2*, *37*). Arg^295^ mutations increased the median effective concentration of both glycine and ivermectin activation by about 10-fold. While α1 Q250C and α1 Q250W had only a small effect on ivermectin currents, α1 Q250E is a hyperekplexia mutation that produces spontaneous currents, and mutant cycle analysis demonstrated that this is due to enhanced interactions with α1 Arg^295^. The position of the equivalent arginine in GABA_A_R has been connected to the efficacy of PAMs that bind the same pocket as ivermectin (*34*). We previously showed that GlyRα1β-20Ivm1000Gly had comparable ivermectin density at each of the α1/α1 and α1/β interfaces, and MD simulations showed similar binding affinity to ivermectin at the α1/β site in the ivermectin/glycine conformation to α1/α1 (*28*). However, without the reinforcement of the desensitized state by glycine, the difference between a glycine and glutamine at the α1 Q250 position may affect ivermectin occupancy at the α1/β interface.

The key factor to weaker binding at the β/α1 interface is the side-chain volume at position β Ile^333^ (α1 Ala^312^), which is located on the M3 helix of the primary subunit. It has previously been shown that the side-chain volume at this position strongly and negatively correlates with ivermectin potency and ivermectin binding affinity (*25*, *28*). At the β/α1 interface, the ivermectin density in GlyRα1β-2Ivm200Stry or GlyRα1β-20Ivm200Stry is weaker compared to similar densities at α1/α1 interfaces. The weak density makes it difficult to resolve the entire ivermectin pose for GlyRα1β-2Ivm200Stry, but, for GlyRα1β-20Ivm200Stry, the density indicates a 1-Å shift of ivermectin’s C12 atom away from the C-α position of α1 Ala^312^ or β Ile^333^ (C12 is within the lactone ring labeled in Figs. 1 and 3). This is similar to what was observed for GlyRα1β-20Ivm1000Gly, where the ivermectin density at the β/α1 interface is as clearly resolved as densities at the α1/α1 and α1/β interfaces but in a subtle yet distinct pose to accommodate the β-specific isoleucine (*28*). MD simulation also showed that binding was weaker at this interface.

The described interactions help rationalize why the α1/β and β/α1 interfaces have lower occupancy than the α1/α1 interfaces. Within the resolution limits, the same interactions are present in GlyRα1β-2Ivm200Stry and GlyRα1β-20Ivm200Stry as GlyRα1β-20Ivm1000Gly. This leads us to conclude that the different conformational states are related to the occupancy of the ligand rather than a distinct binding mechanism. The lower occupancy may be explained by examining the allosteric rearrangements within the TMD. There is a graded response in the TMD rotation and tilt going from GlyRα1β-2Ivm200Stry to GlyRα1β-20Ivm200Stry and lastly to GlyRα1β-20Ivm1000Gly, as discussed further in the section on allosteric rearrangements. Although subtle, this may reshape the binding pocket and lower the affinity for ivermectin binding at the heteromeric interfaces.

### Strychnine binding

**Fig. 4.**
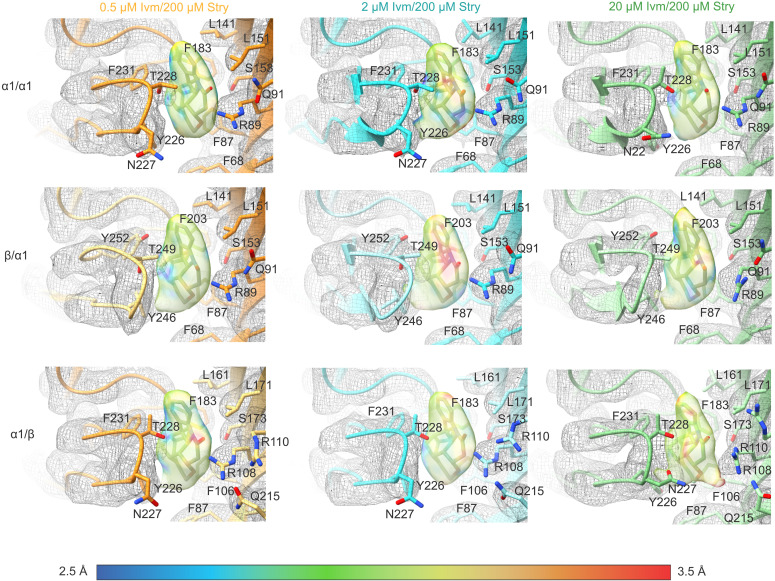
Strychnine binding site. Models and strychnine density are shown for GlyRα1β-0.5Ivm200Stry, GlyRα1β-2Ivm200Stry, and GlyRα1β-20Ivm200Stry at an α1/α1, β/α1, and α1/β interface. Residues important to strychnine binding are shown and labeled, and the strychnine density is colored on the basis of the local resolution as determined by ResMap. The strychnine pose is indistinguishable between GlyRα1β-100Stry, GlyRα1β-0.2Ivm200Stry, GlyRα1β-0.5Ivm200Stry, and GlyRα1β-2Ivm200Stry. In GlyRα1β-20Ivm200Stry, the strychnine density is more diffuse and may be suggestive of pocket instability and/or alternate conformations.

Strychnine binding is observed in all the ivermectin/strychnine structures at the orthosteric binding site (Fig. 4). For GlyRα1β-0.2Ivm200Stry, GlyRα1β-0.5Ivm200Stry, and GlyRα1β-2Ivm200Stry the strychnine pose is indistinguishable from that of GlyRα1β-100Stry (*17*, *28*, *38*). The long axis of the almond-shaped strychnine is mostly perpendicular to the membrane, similar to past findings. The almond tip extends distal to the membrane away from the core of the pocket and contacts α1 Leu^141^ (β Leu^161^). The bulky aliphatic rings of strychnine are surrounded by aromatic GlyR side chains, specifically α1 Phe^183^ (β Phe^203^), α1 Phe^231^ (β Tyr^252^), and α1 Tyr^226^ (β Tyr^246^) from the primary subunit and α1 Phe^87^ (β Phe^106^) from the complementary subunit. The lactam oxygen of strychnine is positioned toward the complementary subunit and interacts with α1 Arg^89^ (β Arg^108^).

The increased ivermectin concentration in GlyRα1β-20Ivm200Stry seems to affect strychnine binding, as the strychnine density is more diffuse and indicative of an alternate binding pose, especially at the α1/β interface. In this structure, Loop C is moved further toward the pocket compared to GlyRα1β-2Ivm200Stry, which constricts the strychnine-binding pocket. This so-called capping of Loop C has been observed across the pLGIC family and generally is associated with agonist binding (*18*) and not observed upon binding of strychnine alone (*17*, *28*). The C-α position of α1 Thr^228^ (β Thr^249^), located on the tip of Loop C, changes by about 4 Å across all five subunits between GlyRα1β-100Stry and GlyRα1β-20Ivm1000Gly. With ivermectin and strychnine, Loop C is still in an overall expanded conformation, but increasing ivermectin concentration incrementally moves the loop toward the capped position. Specifically, the C-α position of α1 Thr^228^ (β Thr^249^) is about 1.5 Å closer to the capped position in GlyRα1β-20Ivm200Stry relative to GlyRα1β-100Stry. This finding reveals that the Loop C capping movement can be triggered by allosteric ligands binding within the TMD, regardless of the nature of the ligand in the binding pocket.

We speculate that further activation may destabilize the pocket and lead to strychnine expulsion entirely, as evidenced by complete removal of strychnine block at higher micromolar ivermectin concentrations (*26*). This is perhaps related to the protective effects of ivermectin against strychnine poisoning (*27*). Consistent with this idea, at the α1/β interface of GlyRα1β-20Ivm200Stry, there is a distinct bulge in the density that faces toward the complementary subunit (Fig. 4). This could result from an alternate conformation of strychnine with the long axis lying horizontally, although we were unable to separate out a distinct cryo-EM class with strychnine in a horizontal conformation. However, strychnine is bound in a horizontal position in crystal structures of acetylcholine binding protein mutated to resemble GlyR, and computational studies also support the possibility of horizontal strychnine orientations (*45*, *46*).

**Fig. 5.**
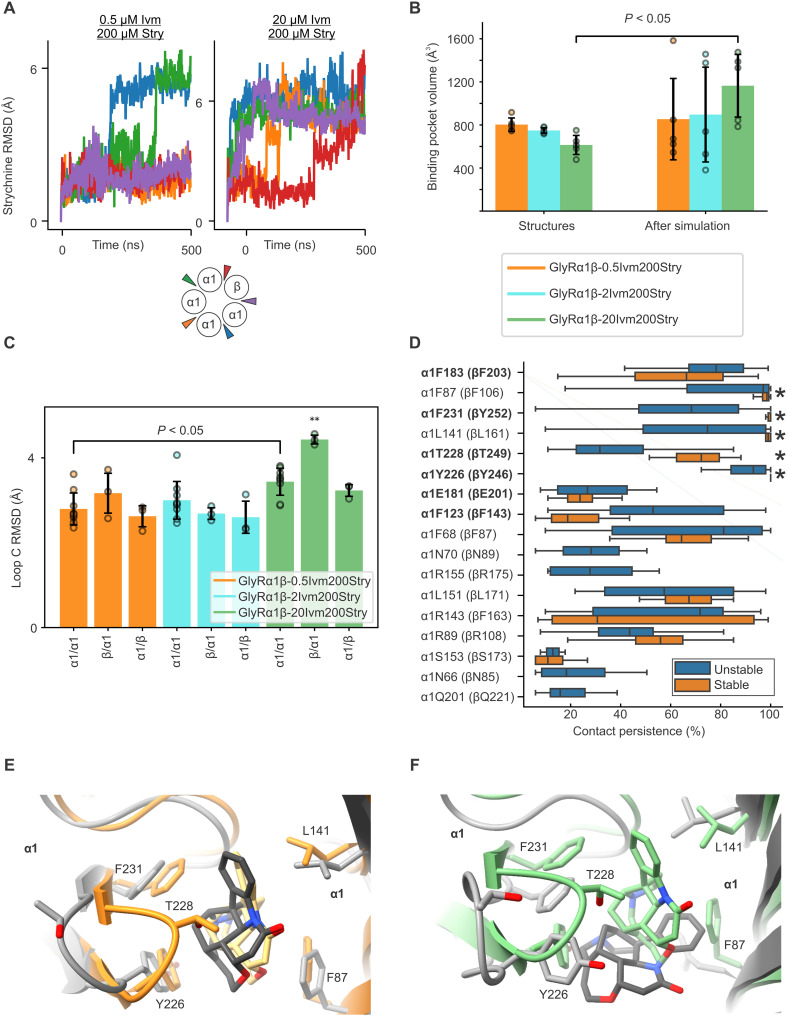
MD simulation of strychnine stability and protein flexibility. Simulations were carried out to assess protein and ligand stability from the starting positions of GlyRα1β-0.5Ivm200Stry, GlyRα1β-2Ivm200Stry, and GlyRα1β-20Ivm200Stry. (**A**) Strychnine root mean square deviations (RMSDs) throughout the simulation show that ligand binding stability is overall higher in GlyRα1β-0.5Ivm200Stry than in GlyRα1β-20Ivm200Stry. The colors correspond to the strychnine positions in the cartoon legend. (**B**) Higher ligand instability correlates with increases in the binding pocket volume. In the case of GlyRα1β-20Ivm200Stry, the pocket volume almost doubled after simulation compared to the initial cryo-EM structure. With *n* = 5 replicates, this difference was significant with *P* = 0.0167. Statistical significance was determined with Welch’s *t* test. Error bars show the SD for each group. (**C**) RMSD plots of Loop C backbone atoms suggest that Loop C movements contribute to the expansion of the binding pocket. Pairwise comparisons were made with Tukey’s post hoc test. Error bars show the SD for each group, and sample sizes and *P* values for significant comparisons are given in table S4. **: The observed difference is statistically different from all other datasets with *P* < 0.05. (**D**) The importance of individual residues to strychnine stability was assessed using contact persistence analysis, which was carried out with ProLIF. This revealed that contacts between strychnine and α1 Phe^87^, Phe^231^, Leu^141^, Tyr^226^, and, to a lesser extent, Thr^228^ (β Phe^106^, Tyr^252^, Leu^161^, Tyr^246^, and Thr^249^) are crucial for stable ligand binding. Primary interface residues are bolded, and the most important interactions for strychnine stability are marked with an asterisk. Examples are given of strychnine molecules that remained stable (**E**) or became unstable (**F**). The starting positions are colored, and the ending positions are gray. While unstable drift occurred in some interfaces of all the tested conditions, simulations starting from GlyRα1β-20Ivm200Stry were about twice as likely to end in an unstable position.

Given the diffuse density and heterogeneity in the GlyRα1β-20Ivm200Stry dataset, we decided to explore the dynamics of strychnine at various subunit interfaces by MD simulation (Fig. 5 and fig. S12). Specifically, simulations were carried out to assess the stability of strychnine binding and the pose of surrounding residues over the course of 500 ns. The initial poses of the receptor and strychnine came from the models of GlyRα1β-0.5Ivm200Stry, GlyRα1β-2Ivm200Stry, and GlyRα1β-20Ivm200Stry. Strychnine was bound at all five interfaces for all three models and ivermectin at all five interfaces for GlyRα1β-2Ivm200Stry and GlyRα1β-20Ivm200Stry. Each simulation was done in triplicate, giving 15 strychnine binding events per condition. In the GlyRα1β-20Ivm200Stry simulations, we chose to start strychnine with the long axis in the horizontal position at the α1/β interface and one of the α1/α1 interfaces (marked as green in the cartoon legend of Fig. 5A). As mentioned, the density was more diffuse at these interfaces, and we rationalized that it was worth exploring the stability of alternate poses (fig. S12A). We do note, however, that the final map and model are best fit with strychnine in the vertical position.

Strychnine root mean square deviation (RMSD) analysis suggests two distinct binding modes. In the first, the ligand remains stably bound with RMSD values at or below 2 Å, and, in the second, strychnine explores other binding modes reaching higher RMSD values of around 6 Å. Across all runs and interfaces, strychnine binding was classified as stable or unstable based on this criterion. A trend was observed, whereby strychnine binding becomes less stable in the simulation for structures solved at increasing ivermectin concentration (Fig. 5A and fig. S12B). Overall, the number of stably bound strychnine molecules decreases going from 10 of 15 (in triplicate simulations) in the GlyRα1β-0.5Ivm200Stry structure to 9 in GlyRα1β-2Ivm200Stry and 1 in the GlyRα1β-20Ivm200Stry. It is worth noting that the alternate binding position used at two interfaces in GlyRα1β-20Ivm200Stry is especially unstable, but the strychnine molecules at the other interfaces show greater instability as well. The unstable strychnine molecules generally drifted so their long axis was horizontal, although there was no clearly defined alternate pose.

Changes in the protein were then assessed to better understand the instability of strychnine in GlyRα1β-20Ivm200Stry. The global RMSD of each system’s protein backbone was calculated and RMSD values of GlyRα1β-20Ivm200Stry simulations are consistently the highest, indicating increased structural flexibility (fig. S12C). Unexpectedly, although the binding pocket volume was smallest for GlyRα1β-20Ivm200Stry in the cryo-EM structure, it nearly doubled during the simulation (Fig. 5B). This final volume was larger than any of the initial cryo-EM structures, and the expansion was not observed with statistical significance in GlyRα1β-0.5Ivm200Stry and GlyRα1β-2Ivm200Stry. There is seemingly a discrepancy in that the volume of the binding pocket for GlyRα1β-20Ivm200Stry is smallest in the cryo-EM model, but largest in the simulation. However, it is critical to note that the MD simulations do not predict a specific structure but simply a more dynamic region with a wider volume. This is consistent with the less defined features and alternate densities in strychnine and the surrounding residues of GlyRα1β-20Ivm200Stry. Similarly, simulations showed the backbone of Loop C in GlyRα1β-20Ivm200Stry had a larger RMSD at the α1/α1 interfaces and the β/α1 interface than the same interfaces in GlyRα1β-0.5Ivm200Stry (Fig. 5C). It may be that this contributes to the instability of strychnine binding observed in simulations (Fig. 5A).

To quantify movements of binding pocket-lining residues, we calculated the root mean square fluctuation (RMSF) of key residues therein (fig. S12D). Expectedly, residues located on Loop C including α1 Tyr^226^, Thr^228^, and Phe^231^ (β Tyr^246^, Thr^249^, and Tyr^252^) show a larger RMSF in GlyRα1β-20Ivm200Stry compared to low-ivermectin conditions. The β hairpin formed by β5-β6 was identified as another region of increased flexibility, as shown through α1 Leu^141^, Arg^143^, and Leu^151^ (β Leu^161^, Phe^163^, and Leu^171^). We conclude that these regions may have a particular role in allosteric communication with the ivermectin binding site. Protein-ligand interaction fingerprints throughout the trajectories were obtained with ProLIF, and the percentage of simulation frames in which an interaction between strychnine and a particular protein residue (referred to as contact persistence) was calculated (*47*). Simulation data from all strychnine binding sites were pooled and split into two groups on the basis of ligand binding stability, and the distributions of contact persistence by ligand stability were compared (Fig. 5D). Stable ligand binding is characterized by persistent contacts to α1 Phe^87^, Phe^231^, Leu^141^, Tyr^226^, and, to a lesser extent, Thr^228^ (β Phe^106^, Tyr^252^, Leu^161^, Tyr^246^, and Thr^249^), again highlighting the importance of Loop C and the β5-β6 hairpin. Findings from mutagenesis and functional studies underscore the importance of each of these side chains in binding strychnine or other orthosteric ligands (*48*–*50*). As these contacts are lost, strychnine explores the surroundings in the binding site more fully, leading to new interactions that are less stabilizing and less likely to contribute to channel modulation. These residues and the strychnine orientation are shown in example frames from the trajectories in Fig. 5 (E and F).

### Allosteric rearrangement of subunit domains

**Fig. 6.**
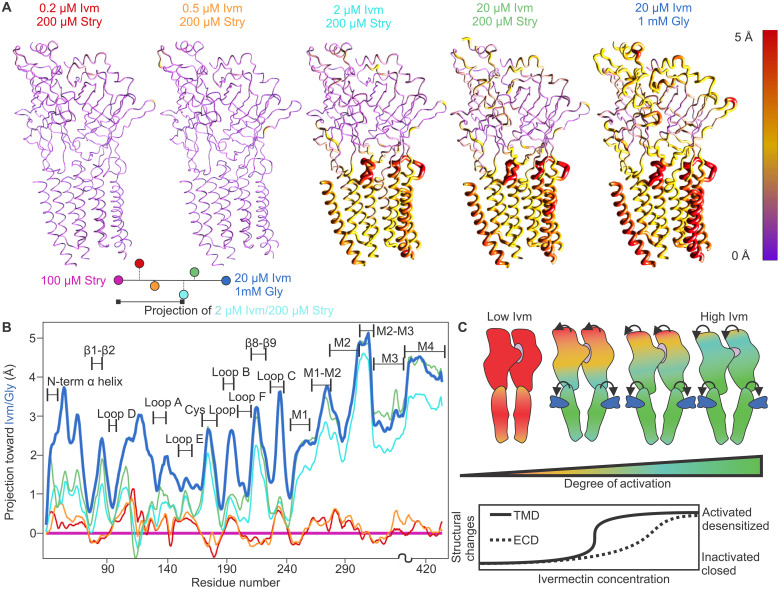
Alignment of strychnine ivermectin structures. (**A**) Heatmaps show the projection of C-α atoms from the closed state (GlyRα1β-100Stry) toward the fully desensitized state (GlyRα1β-20Ivm1000Gly). Plots shown are for the α1/β interface, although results are similar across the receptor. The diagram on the bottom left gives an example of the projection distance being measured. These maps show that increasing ivermectin leads to a coordinated and near-symmetric transition within the TMD from a closed state to a fully desensitized state. The transition within the ECD is more gradual, however, representing a state intermediate to the two extremes that moves toward GlyRα1β-20Ivm1000Gly with increasing ivermectin. (**B**) The same information from (A) but given as a line graph. The coordinated shift of the TMD is apparent starting with the first transmembrane helix onward. (**C**) Cartoon representations summarizing the effect of increasing ivermectin on the ECD and TMD of GlyRα1β. The gray shapes represent strychnine, and the blue shapes ivermectin. Arrows indicate the movements induced by the presence of ivermectin, and increasing the ivermectin concentration induces further movement as indicated by larger arrows. Experimental data represent equilibrium positions using counteracting ligands, and we propose that the transient states of the ivermectin activation pathway follow a similar trajectory in a bottom-up manner.

As described in previous work, differences between the closed GlyRα1β-100Stry and desensitized GlyRα1β-20Ivm1000Gly can be largely described as rigid translations and rotations of the ECD and TMD (*28*). A similar pattern of rigid translations and rotations is seen within the ivermectin/strychnine structures, although the degree of rotation and translation differs between domains. A comparison of the structures shows that increasing ivermectin concentration drives a concerted change within the TMD, while the response is more graded within the ECD where the influence of strychnine seems to limit the degree of activation (Fig. 6 and fig. S13). Although the changes are similar to those induced by orthosteric agonists, it is important to note that ivermectin directly widens the gap between the primary and complementary interface by interacting with residues on the primary M3 and complementary M1 helices. This widening opens the extracellular end of the channel pore and also moves the M2-M3 loop. This initiates the allosteric changes through the ECD/TMD to the rest of the ECD as described in the introduction, but, notably, instead of a conformational wave that starts at the orthosteric site and extends toward the TMD, this wave starts at the allosteric site and transitions from the TMD toward the ECD.

Within the TMD, the helices in GlyRα1β-20Ivm1000Gly displace outward and move counterclockwise relative to GlyRα1β-100Stry. This can be quantified by comparing the center of mass (COM) of atoms within each individual subunit TMD across conformations. The smallest subunit COM shift between GlyRα1β-100Stry and GlyRα1β-20Ivm1000Gly is 2.8 Å (fig. S13A). This analysis for the ivermectin/strychnine structures shows a large conformational change between the closed and desensitized states. The TMD COMs for GlyRα1β-0.2Ivm200Stry and GlyRα1β-0.5Ivm200Stry are within 0.3 Å of their GlyRα1β-100Stry counterparts. Similarly, the TMD COMs of GlyRα1β-2Ivm200Stry and GlyRα1β-20Ivm200Stry are all within 0.6 Å of GlyRα1β-20Ivm1000Gly. The separation into two distinct groupings is consistent with similarities in the pore region and ivermectin binding regions among the observed structures. In addition to changes in the COM, the TMD of each subunit also undergoes a tilt and rotation relative to an axis mostly perpendicular to the membrane and rearrangements can be seen within the orthosteric binding site and at the ECD/TMD interface (fig. S13B). The subunit TMDs without ivermectin bound are hardly tilted or rotated compared to GlyRα1β-100Stry, with average tilt and rotation values across all subunits of 0.2° and 0.4° for GlyRα1β-0.2Ivm200Stry and 0.2° and 0.3° for GlyRα1β-0.5Ivm200Stry. By contrast, the ivermectin-bound subunit TMDs are on average tilted 5.7°, 6.3°, and 7.0° and rotated 12.8°, 13.4°, and 14.2°, respectively, for GlyRα1β-2Ivm200Stry, GlyRα1β-20Ivm200Stry, and GlyRα1β-20Ivm1000Gly. The differences within the two groupings are subtle and altogether support an abrupt, symmetric transition of the TMD with increasing ivermectin concentration.

There is very little change in the ECD COM between the observed structures, with the greatest COM subunit shift being 0.3 Å between GlyRα1β-100Stry and GlyRα1β-20Ivm1000Gly. Instead, global ECD conformational changes can be described as a tilt and rotation of individual subunits about an axis perpendicular to the membrane. Comparing GlyRα1β-100Stry to GlyRα1β-20Ivm1000Gly, the fully desensitized state has an average subunit tilt of 4.3° and rotation of 6.3°. GlyRα1β-0.2Ivm200Stry and GlyRα1β-0.5Ivm200Stry show essentially no changes in their position relative to GlyRα1β-100Stry with average tilt and rotation values of 0.2° and 0.1° for GlyRα1β-0.2Ivm200Stry and 0° and 0.2° for GlyRα1β-0.5Ivm200Stry. GlyRα1β-2Ivm200Stry and GlyRα1β-20Ivm200Stry show an intermediate response to ivermectin activation. GlyRα1β-2Ivm200Stry has an average tilt and rotation value of 3.0° and 0.8°, and GlyRα1β-20Ivm200Stry has values of 3.5° and 1.7°.

The graded response in the ECD suggests that strychnine antagonism resists the allosteric activation by ivermectin. Intuitively, the portions of the ECD that are most involved in strychnine binding will be more resistant to change than those further from the orthosteric binding site. Similarly, the ECD portions of the ECD/TMD interface (M2-M3, β1-β2 loop, Cys-loop, and β8-β9 loop) would be expected to occupy positions most similar to the fully desensitized state. To visualize this effect, a projection value was defined as the distance along a line from a C-α position in GlyRα1β-100Stry to the equivalent C-α in GlyRα1β-20Ivm1000Gly. Figure 6A shows a heatmap marking the TMD transitions that occur more abruptly with increasing ivermectin concentration to the desensitized state, while different regions of the ECD respond individually to increasing ivermectin concentration. Figure 6B shows the same information, but as a line graph that allows one to easily visualize changes within subdomains. For GlyRα1β-2Ivm200Stry and GlyRα1β-20Ivm200Stry, both visualizations show that regions near the ECD/TMD interface are closer to GlyRα1β-20Ivm1000Gly than the middle and distal portions of the ECD. There is also an apparent gradation in the ECD/TMD going from GlyRα1β-2Ivm200Stry to GlyRα1β-20Ivm200Stry, suggesting that the increased ivermectin concentration drives greater changes in these regions (fig. S14).

The regions that make up the orthosteric binding pocket, Loops B and C for example, transition more gradually and are the most distinct between strychnine/ivermectin structures and GlyRα1β-20Ivm1000Gly. Even subtle changes are seen near the orthosteric binding site in GlyRα1β-0.2Ivm200Stry and GlyRα1β-0.5Ivm200Stry. The regions that appear to be the least responsive to ivermectin are those that make up the cleft between subunit interfaces distal to the membrane from the orthosteric binding site. This region is known to bind tricyclic sulfonamide allosteric modulators as part of a mechanism of increasing the channel open probability (*51*, *52*). Figure 6C gives a cumulative model of GlyR allosteric modulation by ivermectin where the TMD moves in a largely symmetric manner and responds in a concerted manner to an increasing ivermectin concentration, while the ECD responds more gradually with different regions being more influenced by the orthosteric or ivermectin allosteric site.

## DISCUSSION

This work demonstrates an allosteric mechanism of GlyR activation and explores interplay between the orthosteric binding site and a physiologically relevant allosteric binding site. We demonstrate that ivermectin, an allosteric agonist, evokes the same structural changes as glycine and other orthosteric agonists but with structural changes originating at the TMD and propagating to the ECD as opposed to acting from ECD to TMD. Although this is intuitive and supported by other recent work, it is not necessary that ivermectin would act along the same pathway (*16*). Obtaining structures at multiple concentrations also demonstrates the regions that form the allosteric network and are most directly connected to the orthosteric or allosteric binding site. In this case, our results suggest that ivermectin activation stabilizes symmetric equilibrium states in a coordinated and symmetric transition of the TMD (*15*). That said, there have been other recently published structures of heteromeric GlyR with an asymmetric M2 conformation, which suggests that, at least, in some cases, gating occurs in a sequential asymmetric manner as has been proposed by MD simulation (*9*, *32*, *33*, *38*).

The ivermectin binding site has been functionally and structurally explored in other pLGICs such as GluCl, nAChR, and GABA_A_R (*16*, *18*, *31*, *34*). Ivermectin acts as a more potent agonist for GluCl, and the crystal structure of GluCl with ivermectin shows a desensitized channel, similar to that observed in GlyR. The diversity of ligands that bind this allosteric site and the pLGIC heterogeneity produce differential effects (*25*). In the case of GlyR, small molecules such as *n*-alcohols and anesthetics such as propofol act at this site, with molecules of different size having either potentiating or inhibitory effect. Across pLGICs, endogenous lipids and other compounds bind the ivermectin pocket, and their efficacy is largely determined by the ability of the molecule to penetrate toward M2 and the accompanying side-chain rearrangements (*31*, *53*). For example, recent related work shows abamectin binds to honey bee GABA_A_R and induces a nonconducting conformation (*53*). The M1/M3 pocket is wide like that of past ivermectin bound structures, but bulky residues in the primary interface push the abamectin toward the complementary interface. The resulting pore is in a conformation that likely precedes channel opening where the extracellular portion of M2 is less tilted than an open/desensitized state and Leu^9^′ is rotated toward the pore lumen. Relevant to our work, this seems to suggest that some degree of channel activation is required for ivermectin to bind, but the effect on the pore depends on the positioning of ivermectin either toward the primary or complementary interface and the molecule’s penetration toward the primary M2 helix. This is consistent with mutagenesis studies that show that bulky side chains in M2 or M3, such as β Ile^333^, reduce ivermectin binding, potency, or even convert it from a positive to a negative allosteric modulator (*25*, *28*).

The orthosteric binding site is also affected by the presence of ivermectin. This is expected as it has been shown that increasing ivermectin concentrations reduced the efficacy of strychnine inhibition (*26*). On the other hand, previous work has suggested that allosteric modulators that bind within the TMD do not affect orthosteric ligand occupancy but rather simply affect the open/closed transition (*16*). Both strychnine and ivermectin can simultaneously bind, suggesting that there is some degree of independence. However, as functional experiments with ivermectin and strychnine show, there is cross-talk between the allosteric and orthosteric sites as GlyRα1β-2Ivm200Stry and GlyRα1β-20Ivm200Stry have partial or distorted ivermectin and strychnine occupancy, respectively. The side chains that make up the binding site are well conserved between the α1 and β, and, without the influence of ivermectin, ligand binding is mostly symmetric (*28*). However, the GlyRα1β-20Ivm200Stry structure demonstrates that asymmetric binding within the ivermectin binding site can differentially affect the subunits and, thus, induce conformational changes between different orthosteric sites, despite the residues being highly conserved. Together, these findings suggest that the allosteric ligands that bind the ivermectin pocket act along a similar pathway as orthosteric ligands, but acting from the TMD to the ECD as opposed to orthosteric ligands that act from the ECD to the TMD.

One goal of this work was to use counteracting ligands to understand the transitions that occur during channel activation. It is important to note that, as the cryo-EM structures represent equilibrium states, they do not forcibly represent transition intermediates. However, the changes observed between structures do allow us to infer the energetics of conformational changes and determine which regions are most responsive to binding at one ligand binding site or another. As an example, one such site is the cleft that sits above the orthosteric site, which remains mostly unchanged with increasing ivermectin concentration. This site has been implicated in zinc modulation of GlyR and is also the binding site of a PAM from Amgen that increases the open-channel probability (*51*, *52*, *54*, *55*). On the basis of work, we propose that, because this site is the last to change from the bottom-up ivermectin activation pathway, it would be among the first in the top-down glycine pathway. Binding of zinc or the Amgen compound could thus facilitate channel activation by lowering this initial energetic hurdle. A more general observation is that the ECD moves more gradually than the TMD. From the top-down perspective, this may mean that there is a set of graded and possibly asymmetric responses within the ECD before activating near-symmetric response from the channel pore, which is in line with other pLGIC studies (*7*, *15*).

Historically, there have been many studies that seek to better understand domain coupling by studying pLGIC transition states that are inferred from electrophysiology models but difficult to directly observe. These include assessing the coupling between residues by measuring the ΔΔ*G* of paired residues, measuring rate constants, and voltage-clamp fluorometry to assess whether a specific residue is closer to one end state or the other during the transition (*1*, *6*, *19*). The results of these studies describe the aforementioned conformational wave in which regions closest to the binding site transition first, then those at the ECD/TMD interface, and lastly the channel pore (*6*, *19*). Our work demonstrates that this wave can also go in the opposite direction from the TMD to the ECD. The principle of signaling from the intracellular or membrane space has been well established in both pLGICs and other protein families such as GPCRs (*22*, *56*). Quaternary changes have been assessed by MD simulation and electrophysiology using combinatorial expression of subunits with defective orthosteric binding sites and/or ECD/TMD coupling regions (*13*, *15*). These show that ligand binding and ECD conformational changes can happen asynchronously among subunits. However, disrupting coupling reduces the channel mean open time while not affecting the conductance, again supporting that channel activity undergoes a concerted and near-symmetric transition of all five subunits within the pore. Some more recent work adds nuance to this view, suggesting that the coupling region and even the pore can move asymmetrically in “primed” states prior or during channel activation (*7*, *32*). The findings of this work suggest that, at least with respect to heteromeric GlyR and ivermectin, the primed states are quite transient and there is a coordinated transition to a near-symmetric desensitized state. Together, this work uses a strategy of counteracting ligands to probe the activation of GlyR by the allosteric agonist ivermectin. This contributes to our understanding of GlyR but also expands the general understanding of the conformational landscape available to pLGICs as they undergo functional transitions. This will, in turn, improve our capacity to modulate these channels and provide better pharmacological tools across the pLGIC family.

## MATERIALS AND METHODS

### Electrophysiological recordings by TEVC

The pCS2-α1 plasmid for expression of ZF GlyRα1 in *X. laevis* oocytes was provided by R. Vandenberg, University of Sydney, Australia, and the pCS2-βb and pCS2-Geph-E constructs for expression of ZF GlyRβb and gephyrin domain E were prepared by GenScript. To linearize the DNA, the plasmids were incubated with Nde I restriction enzyme at 37°C for 2 hours. The mRNA was synthesized from linearized DNA using the mMessage mMachine kit (Ambion) per the instructions in the manufacturer’s manual. The RNA was then purified with the RNeasy kit (QIAGEN). Between 0.4 and 1.6 ng of GlyRα1 mRNA and 3x GlyRβb and Geph-E mRNA were injected into *X. laevis* oocytes (stages V to VI), and experiments were performed 1 day after injection. The oocytes used in this study were purchased from Xenopus 1. Oocytes were maintained at 18°C in frog Ringer’s solution (96 mM NaCl, 2 mM KCl, 1 mM MgCl_2_, 1.8 mM CaCl_2_, and 20 mM Hepes), which was supplemented with 2.5 mM sodium pyruvate, gentamicin (50 μg/ml), and tetracycline (100 μg/ml), with pH adjusted to 7.5 and osmolarity to 195 ± 5 mosmol. TEVC experiments were performed on an Axon Instruments Axoclamp 900A. Currents were sampled and digitized at 500 Hz with an Axon Digidata 1550B and analyzed by Clampfit 10.7.0.3/11.4.2 (Molecular Devices). Oocytes were clamped at a holding potential of −60 mV, and solutions were exchanged using a syringe pump perfusion system flowing at a rate of ~10 ml/min. The electrophysiological solutions consisted of 96 mM NaCl, 2 mM KCl, 1.8 mM CaCl_2_, 1 mM MgCl_2_, and 20 mM Hepes (pH 7.5, osmolarity adjusted to 195 ± 5 mosmol). Glycine, ivermectin, and strychnine were all purchased from Sigma-Aldrich. Current traces were plotted using Origin Version b9.5.0.193. All data are reported as means ± SE for (*n*) individual oocytes. No sample size calculation was made. All statistical tests were unpaired and two-sided and summarized in table S1.

### Protein purification for cryo-EM studies

The cloned genes were prepared by GenScript and used in a previous publication (*28*). The GlyRα1 construct has a thrombin cleavage site (LVPRGS) followed by a C-terminal 8xHis tag, and GlyRβb was modified to have a TEV cleavage site (ENLYFQG) followed by a C-terminal Rho-1D4 (TETSQVAPA) antigen-binding site [National Center for Biotechnology Information (NCBI) reference sequence: GlyRα1 NP_571477.1 and GlyRβB NP_001003587.1] (*36*). GlyR genes were also coexpressed with the E domain of gephyrin with an N-terminal flag tag (DYKDDDDK) followed by a poly-asparagine linker and TEV cleavage site (E-Geph, residues 398 to 769, NCBI reference sequence: NP_074056.2). ZF GlyRα1, GlyRβb, and E-Geph have 86, 85, and 100% sequence homology to their human counterparts, respectively. Genes were codon optimized for insect cell expression and cloned into a pFastBacDual vector with GlyRα1 on the polyhedrin promoter and GlyRβb on the p10 promoter. The Geph-E gene was similarly codon optimized and cloned into a pFastBac1 vector. Baculovirus was generated and protein was produced in ExpiSf9 cells (Invitrogen). Cells were harvested 48 to 72 hours postinfection, pelleted, and resuspended in lysis buffer [20 mM tris base, 36.5 mM sucrose, 10% glycerol, and 0.25% Sigma 8340 protease cocktail inhibitor (pH 8.0)] and then flash frozen. Membranes were prepared from thawed cells and lysed by gentle sonication. The lysed cells were spun at 3200*g* for 15 min to remove large cell debris and then spun for 1 hour at 167,000*g*. The supernatant was discarded, and the membrane pellet was resuspended in membrane buffer [20 mM Hepes, 150 mM NaCl, and 10% glycerol (pH 8.0)] and flash frozen.

The day of purification, membranes were thawed and then solubilized by adding 20 mM *n*-dodecyl-β-d-maltopyranoside (DDM; Anatrace) supplemented with 0.05% cholesteryl hemisuccinate (CHS; Anatrace) and soybean polar extract (0.05 mg/ml; Avanti Polar Lipids) and rotated at 4°C for 2 hours. Nonsolubilized debris was removed by centrifugation for 20 min at 167,000*g*. The resulting supernatant was then bound to 0.5 ml of Rho-1D4 beads (Rho-1D4 antibody conjugated to cyanogen bromide-activated sepharose beads; Cytiva, 17-0430-01) for every 1 liter of culture (1 ml total) and rocked for 2 hours. The beads were then washed with 10 column volumes (CVs) of GlyR wash buffer (membrane buffer with 1 mM DDM and 0.05% CHS). Protein bound to the Rho-1D4 beads was then eluted using four CVs of GlyR wash buffer supplemented with Rho-1D4 peptide (4 mg/ml; GenScript, TETSQVAPA). After 2 to 6 hours, the first elution was collected and replaced with an additional four CVs of fresh elution buffer. The next morning, the two elution fractions were pooled and then bound to 0.7 ml of preequilibrated Ni-NTA agarose beads (QIAGEN, 30210) at 4°C for 1.5 hours. The beads were washed in GlyR wash buffer with 25 mM imidazole followed by five elution fractions of one CV of GlyR wash buffer with 250 mM imidazole, each separated by 5 min. The elutions were pooled and concentrated to about 0.5 ml using a 100-kDa molecular weight cut-off Millipore filter (Amicon, UCF810024). The concentrated protein was then filtered using a 0.22-μm polyvinylidene difluoride filter and passed through a Superose 6 Increase column (Cytiva, 29091596), preequilibrated with filtration buffer [20 mM Hepes, 150 mM NaCl, and 1 mM DDM (pH 8.0)]. Western blot analysis demonstrated that all three proteins came in the main peak near 14.5 ml. The peak fraction had a protein yield of 0.05 to 0.1 mg/ml. This was not concentrated further as this led to protein degradation as observed on cryo-EM grids.

### Cryo-EM sample preparation

Because of the low concentration, cryo-EM samples were prepared using graphene oxide grids to enhance particle density within grid holes (*57*). This resulted in preferred sample orientation, but the effects were manageable with a large but reasonable amount of data collection (10,000 to 20,000 movies). Ivermectin (Sigma-Aldrich) was prepared at a stock of 40 mM in dimethyl sulfoxide and diluted to 400 μM in filtration buffer. This solution was insoluble but vigorously vortexed immediately before further dilution in the protein solution so that the final concentration was 0.2, 0.5, 2, or 20 μM ivermectin (accounting for volume that would be added from the strychnine stock as well). The sample was then kept on ice for 20 min to allow the slow-acting ivermectin to reach equilibrium and then strychnine HCl (Sigma-Aldrich) was added from a 4 mM stock to 200 μM final concentration. Grids were frozen on Holey Carbon R1.2/1.3 grids (Quantifoil) using a Vitrobot Mark IV (Thermo Fisher Scientific).

### Cryo-EM data acquisition and image processing

Imaging was done at either Case Western Reserve University (CWRU; GlyRα1β-0.2Ivm200Stry, GlyRα1β-0.5Ivm200Stry, and GlyRα1β-2Ivm200Stry) or New York Structural Biology Center (NYSBC; GlyRα1β-20Ivm200Stry) using a 300-kV FEI Titan Krios microscope equipped with a K3 camera and a Gatan Imaging Filter. Leginon at NYSBC and EPU or SerialEM at CWRU were used for automated data acquisition. Datasets were collected in normal counting mode at ×105,000 magnification giving a pixel size of 0.82 Å/pixel at NYSBC and 0.84 Å/pixel at CWRU. The dose rate was 15 to 20 electron (e^−^)/pixel per second, the total dose was 60 e^−^/Å^2^, and the target defocus ranged from −0.8 to −1.6 μm. A total of 8740, 9711, 25,020, and 38,701 movies were collected, respectively, for GlyRα1β-0.2Ivm200Stry, GlyRα1β-0.5Ivm200Stry, GlyRα1β-2Ivm200Stry, and GlyRα1β-20Ivm200Stry. Further details are given in figs. S2 to S9 and table S2.

Image preprocessing was done using the RELION (v. 5.0) pipeline including their implementation of MotionCorr2 (v. 1.2.3) and CTF correction by CTFFIND v4.1 (*58*–*60*). As graphene oxide coating was variable, micrographs were filtered on the basis of the CTF “Figure of Merit” parameter, which generally resulted in micrographs with a thin layer of graphene oxide. Particles were picked using a template from a previous dataset. About 1 to 3 million particles were picked from each dataset, the majority of which were junk that was sorted out through several rounds of 2D classification (figs. S2 to S5). After achieving high quality 2D classes, an ab initio volume was generated using cryoSPARC (v. 4.6.2) and that model was then used as an input for C5 nonuniform (NU) refinement in cryoSPARC (*61*). Although this symmetry is incorrect, this helped get an initial alignment of the particles. This was followed by Bayesian polishing in RELION with transitions aided by the pyem software (*62*). 3D classification was then done in RELION with C5 symmetry relaxation using such tight angles that there was practically no angular refinement aside from symmetry relaxation (*63*). This produced some classes with asymmetric features that were then used for the next round of NU refinement in C1. This was repeated two to three times, and, then, 3D classification was done without image refinement. At each stage, there were more and more particles that aligned to asymmetric C1 positions as determined by examining the expected asymmetric features (*28*). These include subunit-specific glycosylation and N- and C-terminal extensions. After no further improvement was seen in asymmetric features, another round of Bayesian polishing was done in RELION followed by multiple rounds of 3D classification with no image alignment, CTF-Refinement, and local 3D refinement with BLUSH until the final map resolution was achieved (*64*). Notably, BLUSH regularization substantially improved preferred orientation artifacts. The final particle numbers and map resolutions are given in table S2. Local resolution was done using ResMap software, as implemented within RELION (*65*). The ResMap algorithm defines resolution differently from an Fourier shell correlation (FSC) based measure of resolution, but the results are generally consistent with the 0.143 FSC determined global resolutions.

### Model building and analysis of cryo-EM data

Cryo-EM maps were modeled using density maps that were masked but unsharpened. Previously published models of GlyRα1β-100Stry and GlyRα1β-20Ivm21000Gly were used as starting points for closed and desensitized structures, respectively (*28*). The models were then grossly fit using stepped refine and zoned real-space refine in Coot (v. 0.9.8.96) and then refined using phenix real-space refinement in the PHENIX GUI (v. 1.21.2-5419) (*66*). Further iterations in Coot and PHENIX were done to improve map/model correlations and model statistics with final statistics reported from the PHENIX module mtriage and MolProbity (table S2) (*67*, *68*). Maps and models covered most of the ECD and TMD. Although glycosylation sites and N- and C-terminal segments of each subunit were less well resolved, their presence or absence was clear and sufficient for subunit identification. Only the initial segments of the M3-M4 loop, which forms the ICD, were resolved, consistent with other anionic pLGICs (*17*, *18*). Ion channel pore analysis was done using HOLE (v. 3.0) (*39*). Figures were prepared with ChimeraX (v. 1.1) and CorelDraw (v. 23.0.1.389). Principal components analysis (PCA) was done using MATLAB (MathWorks, v. R2018b), as previously described (*28*). First, the atomic coordinates of the subunit domain being interrogated were aligned on the basis of their COM. Then, PCA analysis was done on the coordinates giving six degrees of freedom. The first three PCA vectors essentially corresponded to rigid rotations, while the remaining three corresponded to local deformations. Ligand analysis was done using LigPlot (v 4.5.3).

### MD simulation

The GlyRα1β-0.5Ivm200Stry, GlyRα1β-2Ivm200Stry, and GlyRα1β-20Ivm200Stry systems were considered in MD simulations. The missing ICD in each segment was substituted with a tripeptide linker (AGT) as in previous studies (*29*) using MODELLER (*69*) version 9.25. Each structure was then embedded in a uniform POPE (1-palmitoyl-2-oleoyl-*sn*-glycero-3-phosphoethanolamine) bilayer with the InflateGRO method (*70*) and solvated with TIP3P water (*71*) containing 150 mM NaCl. Simulations were performed with GROMACS 2021.7 (*72*). Protein and lipid were described with the CHARMM36m force field (*73*), while strychnine and ivermectin were modelled with CGenFF (*74*). Bonds to hydrogen were constrained with the LINCS algorithm (*75*). A time step of 2 fs was used for integration of the equations of motion. Long-ranged electrostatic interactions were calculated with a smooth particle mesh Ewald approach (*76*), while short-ranged interactions were calculated under a Verlet cutoff scheme. The V-rescale thermostat (*77*) and C-rescale barostat (*78*) with coupling constants of 1 and 5 ps, respectively, were used to maintain a temperature of 310 K and a pressure of 1 bar throughout the simulation. A careful equilibration protocol was used by which position restraints on protein atoms and lipid headgroups were gradually released. After all other restraints had been lifted, those on the ligand were gradually released in a stepwise fashion. After total equilibration times of 200 ns, unrestrained production simulations of 500 ns were carried out in triplicate.

RMSD and RMSF values were calculated with in-house MDAnalysis (*79*, *80*) scripts. Interactions between ligand and protein were counted with ProLIF (*47*). The contact persistence of an interaction was then determined as the fraction of simulation frames in which it was found by ProLIF.
