# Supplementary material for: Structural basis for domain coupling in heteromeric glycine receptors revealed by an atypical allosteric agonist
Source: Sci Adv. 2026 Feb 13;12(7):eaeb2036. doi: 10.1126/sciadv.aeb2036 (PMC12904182; doi:10.1126/sciadv.aeb2036)
Supplement: Supplementary file 1 — Figs. S1 to S14 Tables S1 to S4 [file sciadv.aeb2036_sm.pdf]

Science Advances
AAAS

Supplementary Materials for

Structural basis for domain coupling in heteromeric glycine receptors revealed by an atypical allosteric agonist

Eric Gibbs *et al.*

Corresponding author: Philip C. Biggin, philip.biggin@bioch.ox.ac.uk;
Sudha Chakrapani, sudha.chakrapani@case.edu

Sci. Adv. **12**, eaeb2036 (2026)
DOI: 10.1126/sciadv.aeb2036

This PDF file includes:

Figs. S1 to S14
Tables S1 to S4

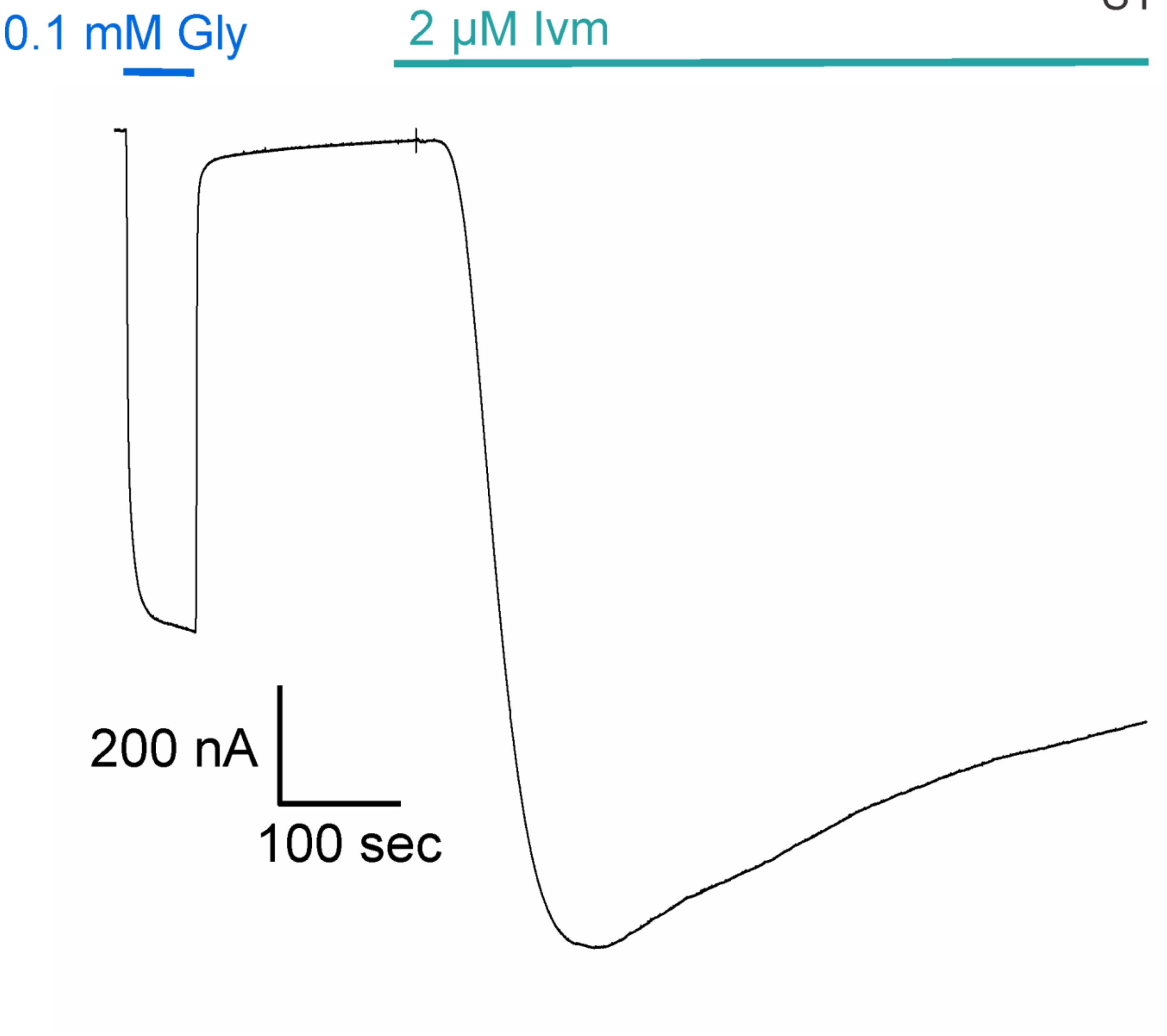

Supplementary Figure 1: Desensitization trace of ivermectin currents.
TEVC current demonstrating the rate of desensitization for ivermectin currents.

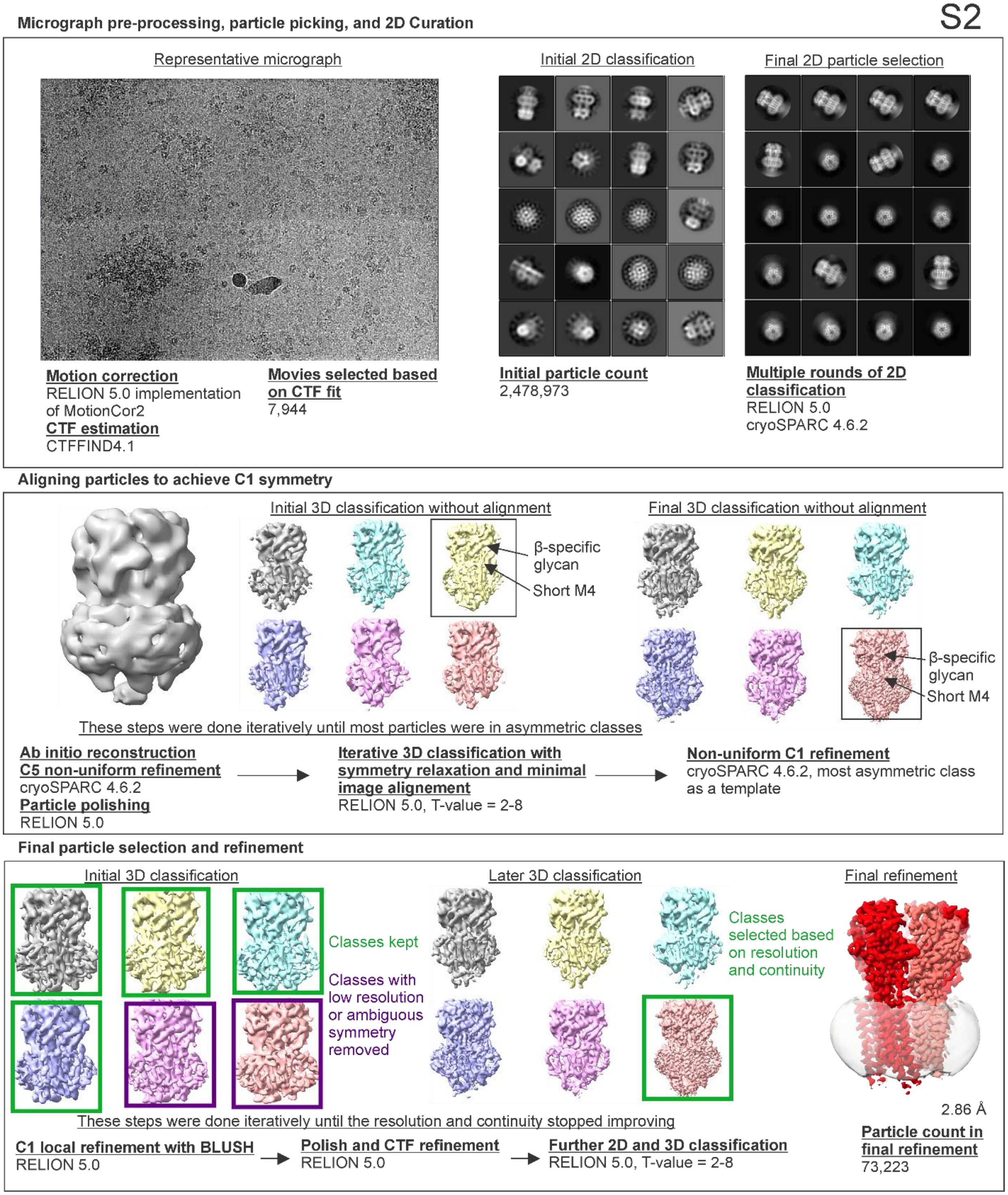

Supplementary Figure 2: Cryo-EM data processing strategy for GlyRα1β-0.2Ivm200Stry.
The data processing strategy can be broken down into three specific parts. The first step involves micrograph treatment and curation by 2D classification. This was done iteratively until high-resolution 2D classes were achieved. The second step was symmetry alignment. This was done by running 3D classification with symmetry relaxation or 3D classification without image alignment and using asymmetric classes as templates. Particles were only removed if they were part of classes that were missing significant portions of the protein. This was done iteratively until nearly all particles were sorted into asymmetric classes. The third step involved Bayesian polishing, CTF refinement, 3D classification without image alignment, and local angular refinement to preserve the asymmetric angular distribution. Particles in classes with poor resolution, continuity, or with ambiguous asymmetric features (for example, α1 and β glycans were present on the same subunit) were removed. This was done iteratively until there was no further improvement in the resolution and continuity of the final map. No clear alternate conformations were observed through the classifications and refinement.

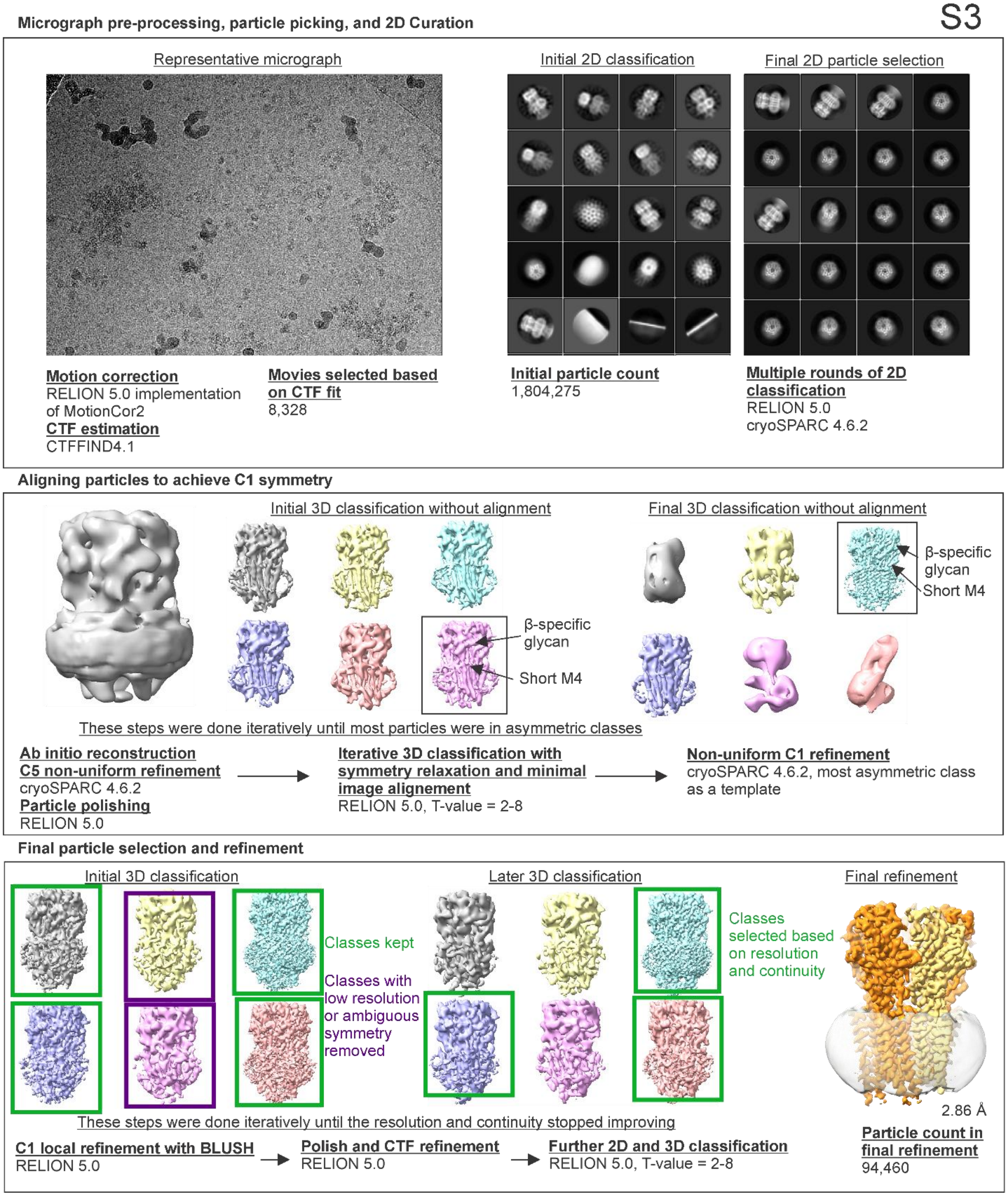

Supplementary Figure 3: Cryo-EM data processing strategy for GlyRα1β-0.5Ivm200Stry.
The data processing strategy can be broken down into three specific parts. The first step involves micrograph treatment and curation by 2D classification. This was done iteratively until high-resolution 2D classes were achieved. The second step was symmetry alignment. This was done by running 3D classification without alignment (no symmetry relaxation was needed in this case) and using asymmetric classes as templates. Particles were only removed if they were part of classes that were missing significant portions of the protein. This was done iteratively until nearly all particles were sorted into asymmetric classes. The third step involved Bayesian polishing, CTF refinement, 3D classification without image alignment, and local angular refinement to preserve the asymmetric angular distribution. Particles in classes with poor resolution, continuity, or with ambiguous asymmetric features (for example, α1 and β glycans were present on the same subunit) were removed. This was done iteratively until there was no further improvement in the resolution and continuity of the final map. No clear alternate conformations were observed through the classifications and refinement.

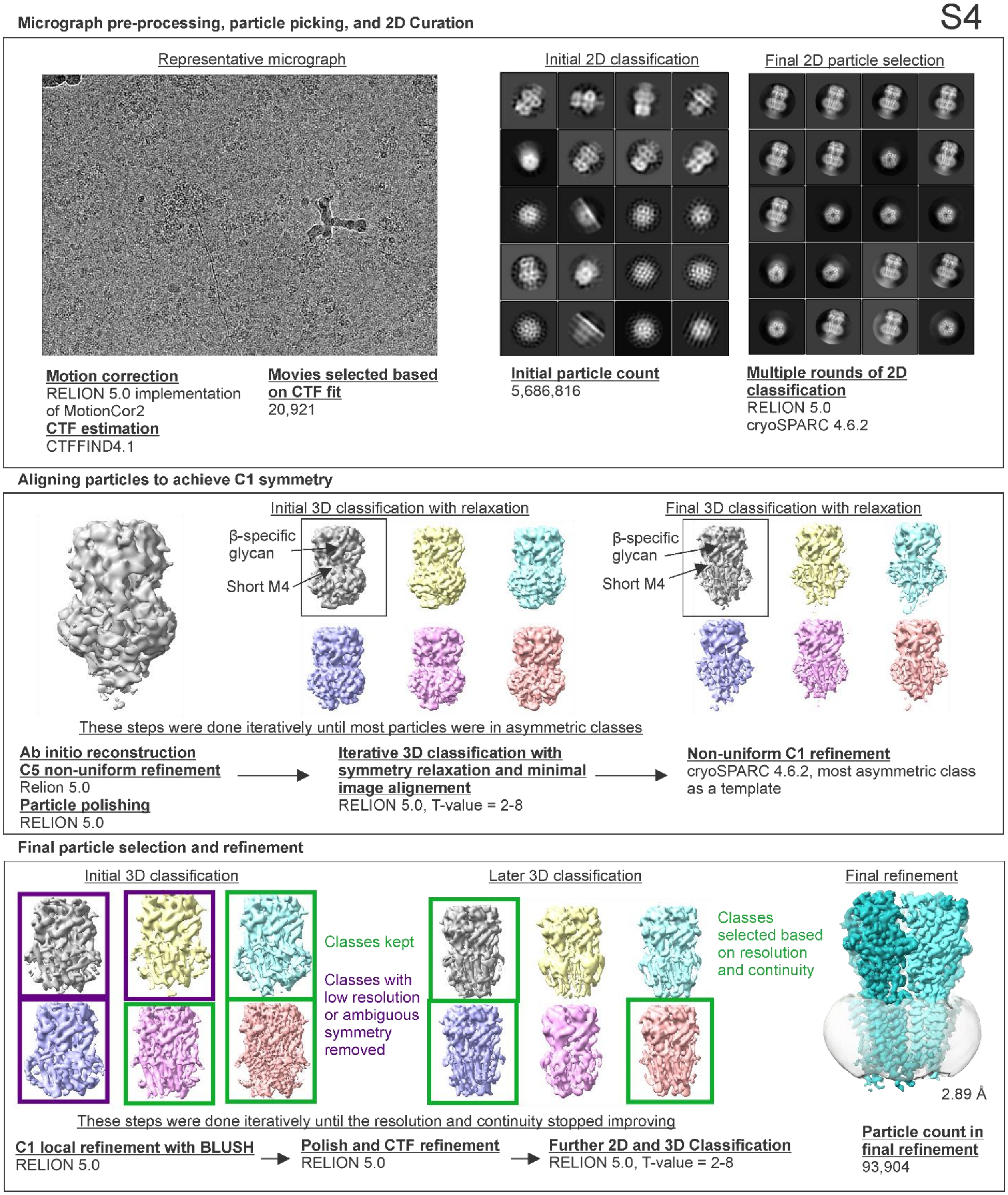

Supplementary Figure 4: Cryo-EM data processing strategy for GlyRα1β-2lvm200Stry.
The data processing strategy can be broken down into three specific parts. The first step involves micrograph treatment and curation by 2D classification. This was done iteratively until high-resolution 2D classes were achieved. The second step was symmetry alignment. This was done by running 3D classification with symmetry relaxation and using asymmetric classes as templates. Particles were only removed if they were part of classes that were missing significant portions of the protein. This was done iteratively until nearly all particles were sorted into asymmetric classes. The third step involved Bayesian polishing, CTF refinement, 3D classification without image alignment, and local angular refinement to preserve the asymmetric angular distribution. Particles in classes with poor resolution, continuity, or with ambiguous asymmetric features (for example, α1 and β glycans were present on the same subunit) were removed. This was done iteratively until there was no further improvement in the resolution and continuity of the final map. No clear alternate conformations were observed through the classifications and refinement.

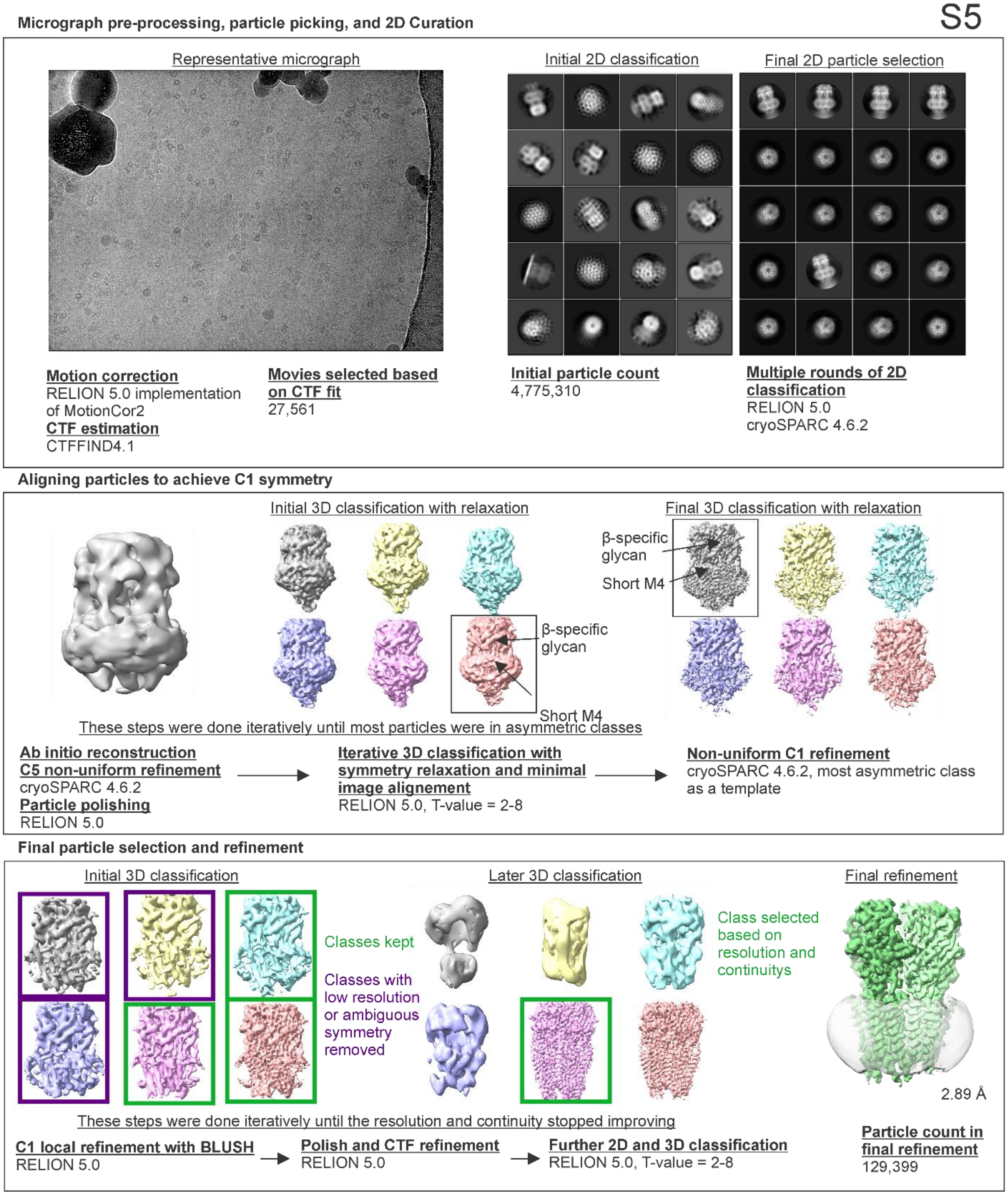

Supplementary Figure 5: Cryo-EM data processing strategy for GlyRα1β-20Ivm200Stry.

The data processing strategy can be broken down into three specific parts. The first step involves micrograph treatment and curation by 2D classification. This was done iteratively until high-resolution 2D classes were achieved. The second step was symmetry alignment. This was done by running 3D classification with symmetry relaxation and using asymmetric classes as templates. Particles were only removed if they were part of classes that were missing significant portions of the protein. This was done iteratively until nearly all particles were sorted into asymmetric classes. The third step involved Bayesian polishing, CTF refinement, 3D classification without image alignment, and local angular refinement to preserve the asymmetric angular distribution. Particles in classes with poor resolution, continuity, or with ambiguous asymmetric features (for example, α1 and β glycans were present on the same subunit) were removed. This was done iteratively until there was no further improvement in the resolution and continuity of the final map. No clear alternate conformations were observed through the classifications and refinement.

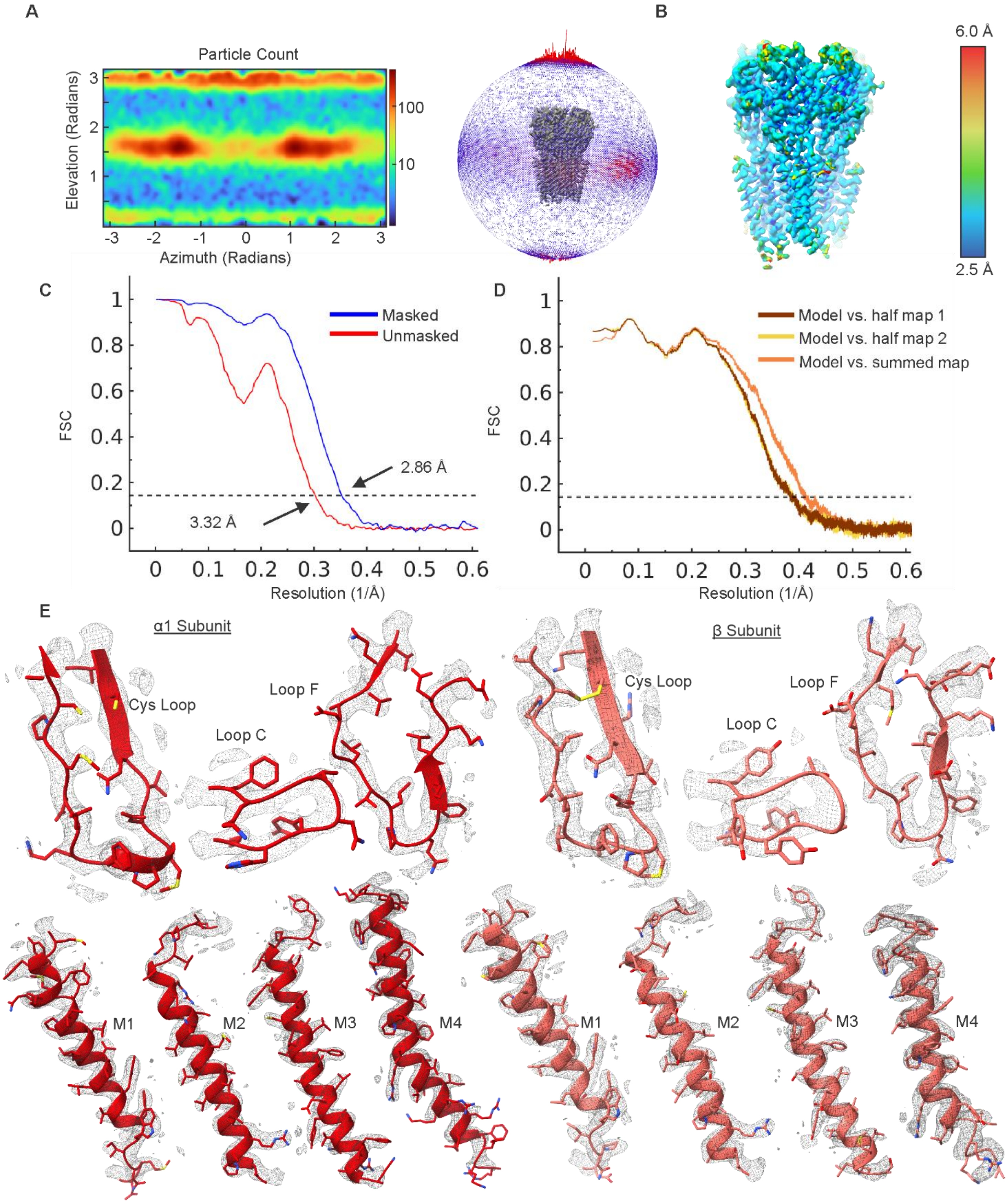

Supplementary Figure 6: Map/model validation of GlyRα1β-0.2Ivm200Stry.

A) Angular distribution maps from particles used for high-resolution 3D image reconstruction. B) Local resolution map generated using ResMap. C) FSC curves generated from a RELION postprocessing job. D) Map/model correlations generated using the PHENIX mtriage routine. E) Models and corresponding map density for various regions for GlyRα1β-0.2Ivm200Stry. The α1 subunit shown is the second clockwise β subunit when viewed from the extracellular space. The map thresholds for each region in both subunits are 0.005 except Loop C, which was 0.008.

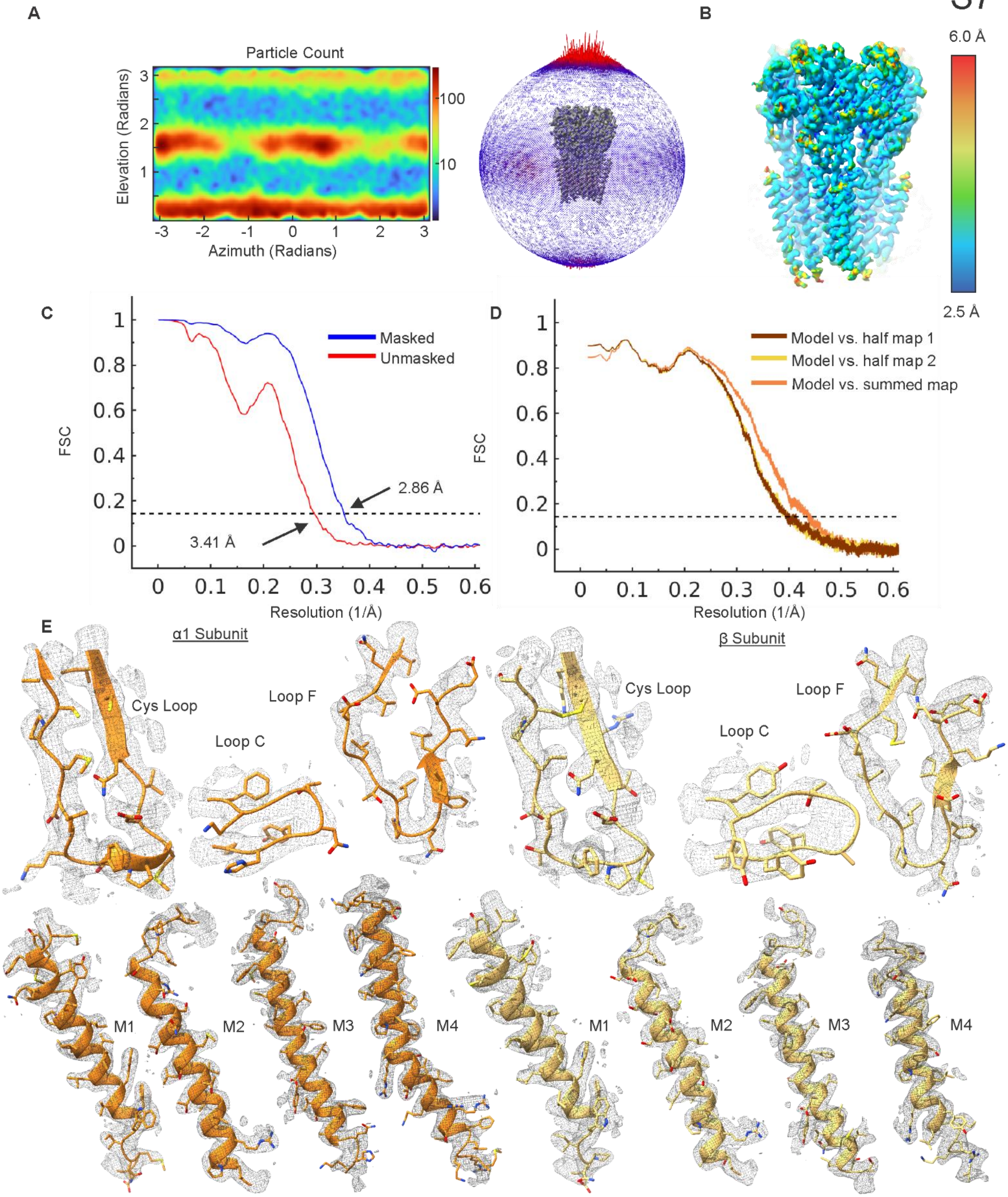

Supplementary Figure 7: Map/model validation of GlyRα1β-0.5Ivm200Stry.

A) Angular distribution maps from particles used for high-resolution 3D image reconstruction. B) Local resolution map generated using ResMap. C) FSC curves generated from a RELION postprocessing job. D) Map/model correlations generated using the PHENIX mtriage routine. E) Models and corresponding map density for various regions for GlyRα1β-0.5Ivm200Stry. The α1 subunit shown is the second clockwise β subunit when viewed from the extracellular space. The map thresholds for each region in both subunits are 0.005 except Loop C, which was 0.008.

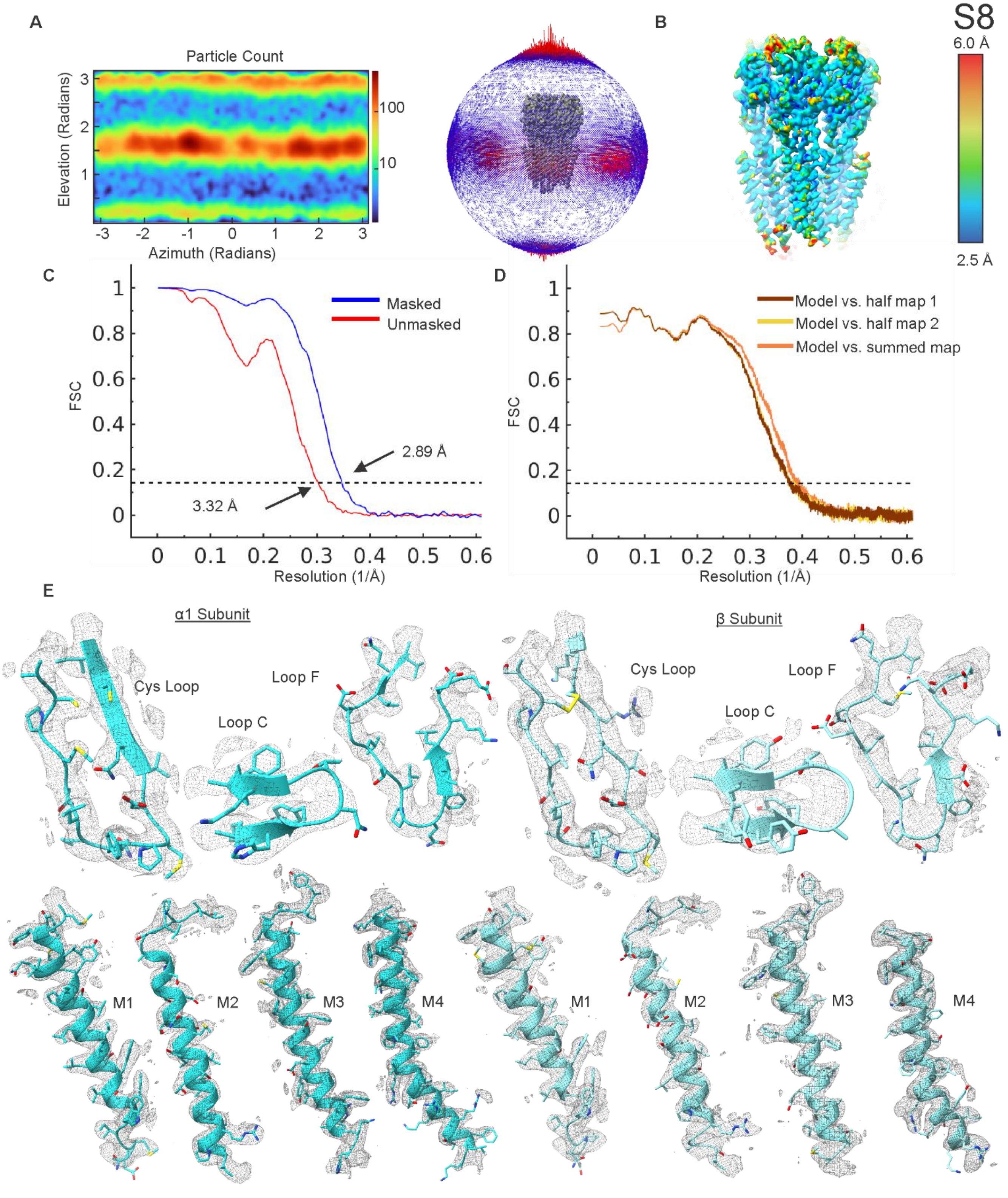

Supplementary Figure 8: Map/model validation of GlyRα1β-2Ivm200Stry.
A) Angular distribution maps from particles used for high-resolution 3D image reconstruction. B) Local resolution map generated using ResMap. C) FSC curves generated from a RELION postprocessing job. D) Map/model correlations generated using the PHENIX mtriage routine. E) Models and corresponding map density for various regions for GlyRα1β-2Ivm200Stry. The α1 subunit shown is the second clockwise β subunit when viewed from the extracellular space. The map thresholds for each region in both subunits are 0.005 except Loop C, which was 0.008.

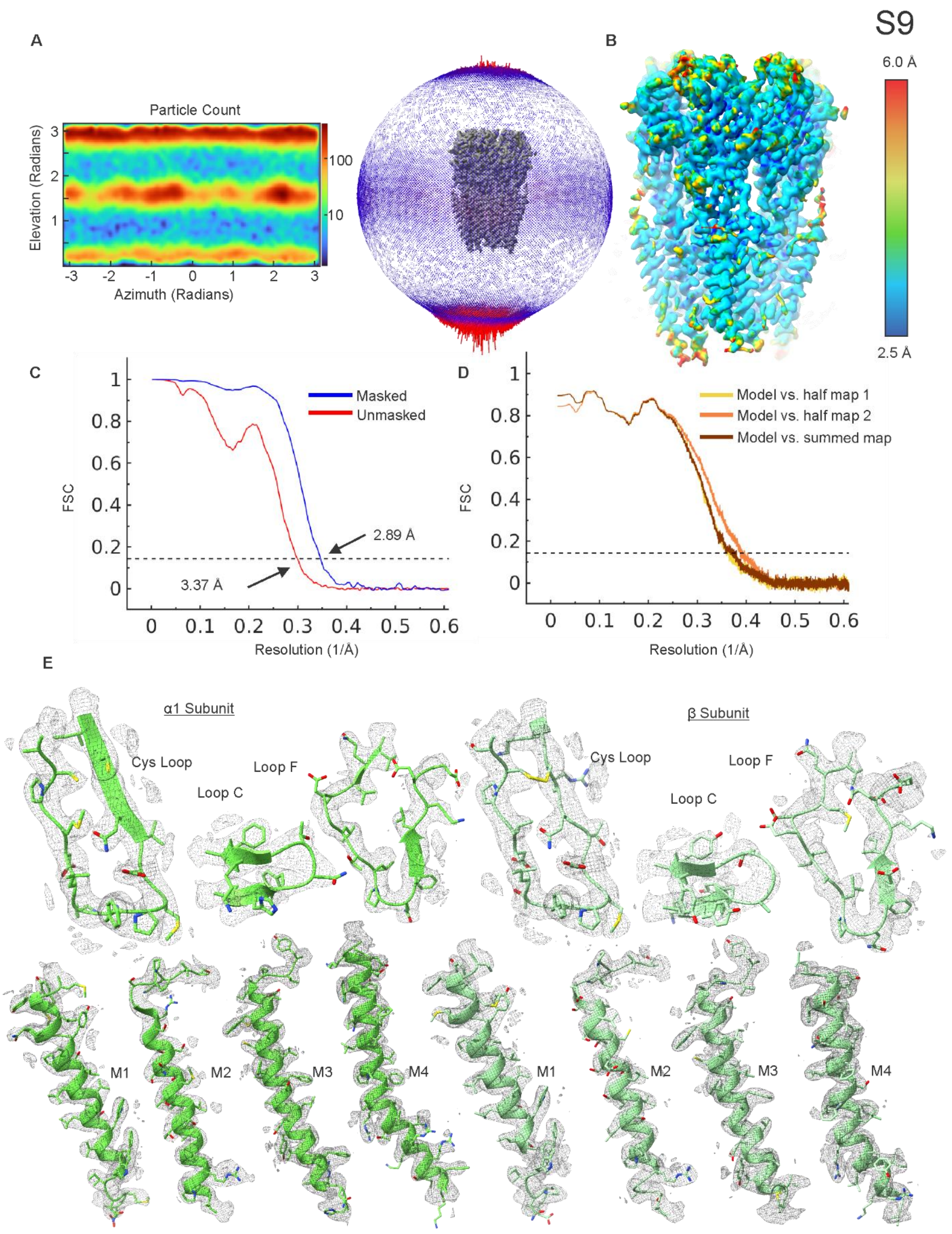

Supplementary Figure 9: Map/model validation of GlyRα1β-20Ivm200Stry.

A) Angular distribution maps from particles used for high-resolution 3D image reconstruction. B) Local resolution map generated using ResMap. C) FSC curves generated from a RELION postprocessing job. D) Map/model correlations generated using the PHENIX mtriage routine. E) Models and corresponding map density for various regions for GlyRα1β-20Ivm200Stry. The α1 subunit shown is the second clockwise β subunit when viewed from the extracellular space. The map thresholds for each region in both subunits are 0.005 except Loop C, which was 0.0065.

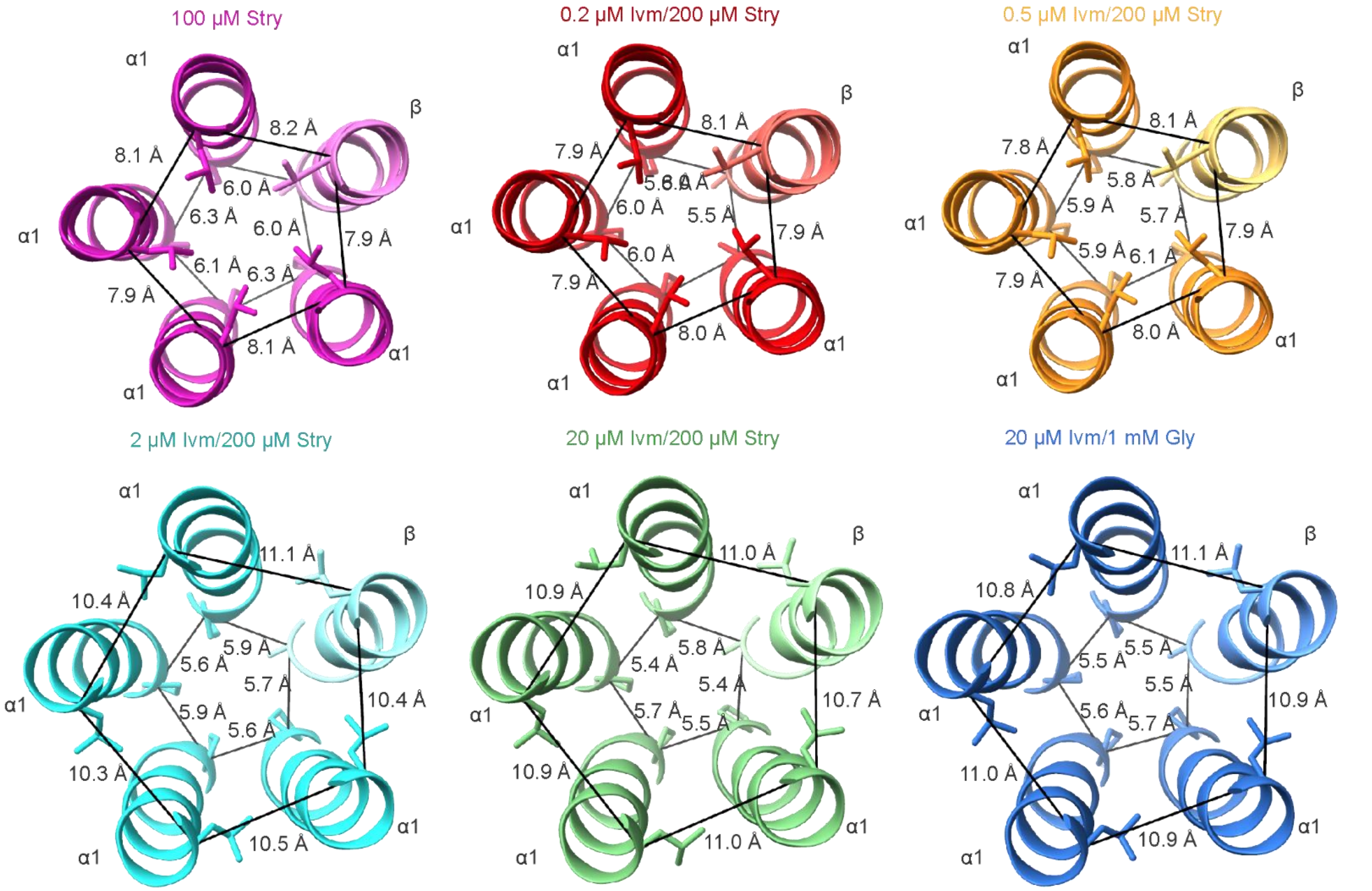

Supplementary Figure 10: Measurements of the pore at the activation and desensitization gates. Images of the pore viewed from the extracellular space towards the cytoplasm. The distances between neighboring C-α atoms are shown at the Leu9′ activation gate (top) and Pro/Ala-2′ desensitization gate (bottom). The pore is generally symmetric and there are minimal differences within the groupings of the closed states (top row) and desensitized states (bottom row).

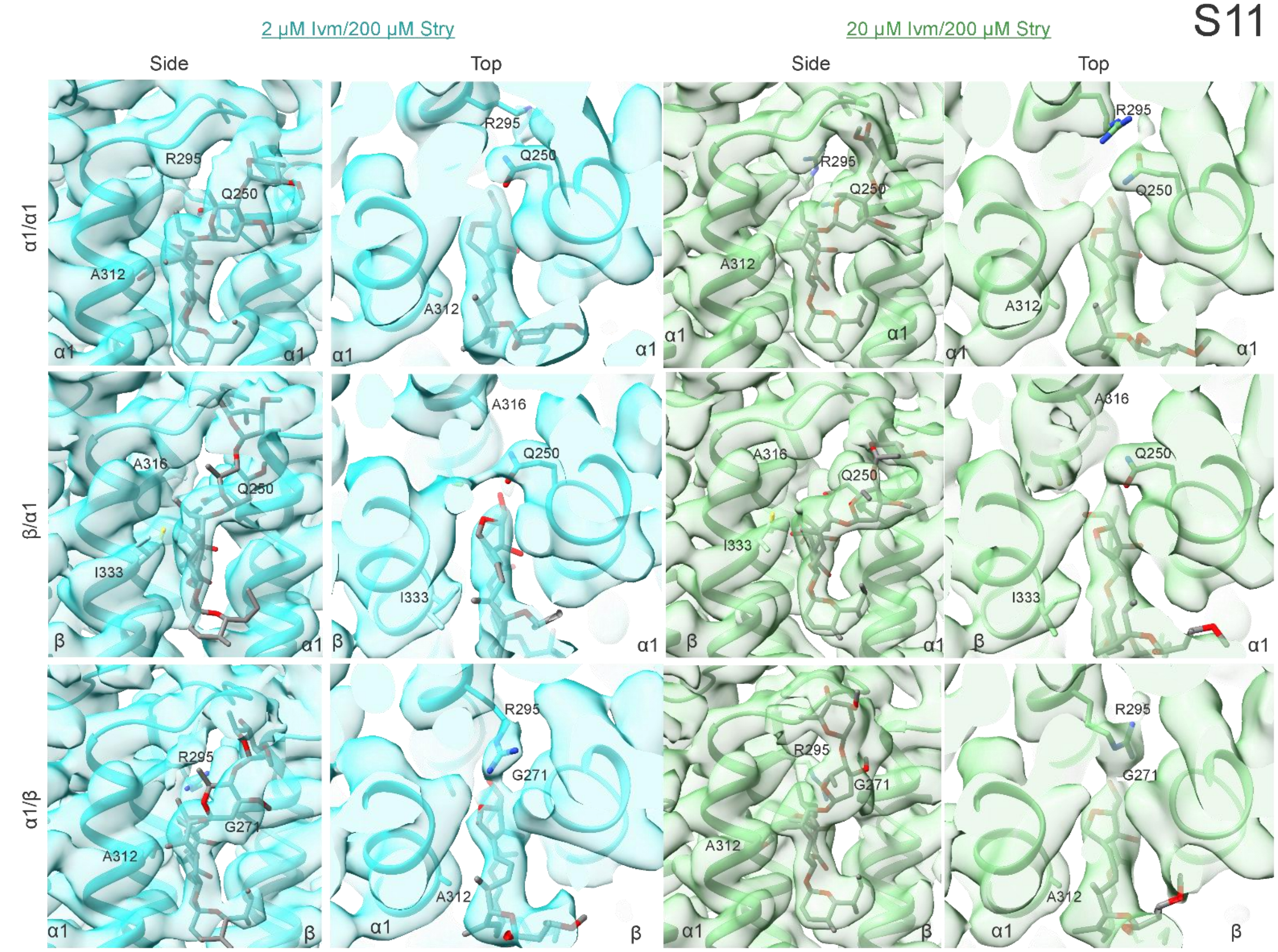

Supplementary Figure 11: Map and model of the ivermectin binding site.
The models for GlyRα1β-2Ivm200Stry and GlyRα1β-20Ivm200Stry are shown as cartoons with side chains shown for residues discussed in the text. Map density is shown as a transparent surface at a threshold value of 0.003. This low threshold allows one to observe density for the side chains shown.

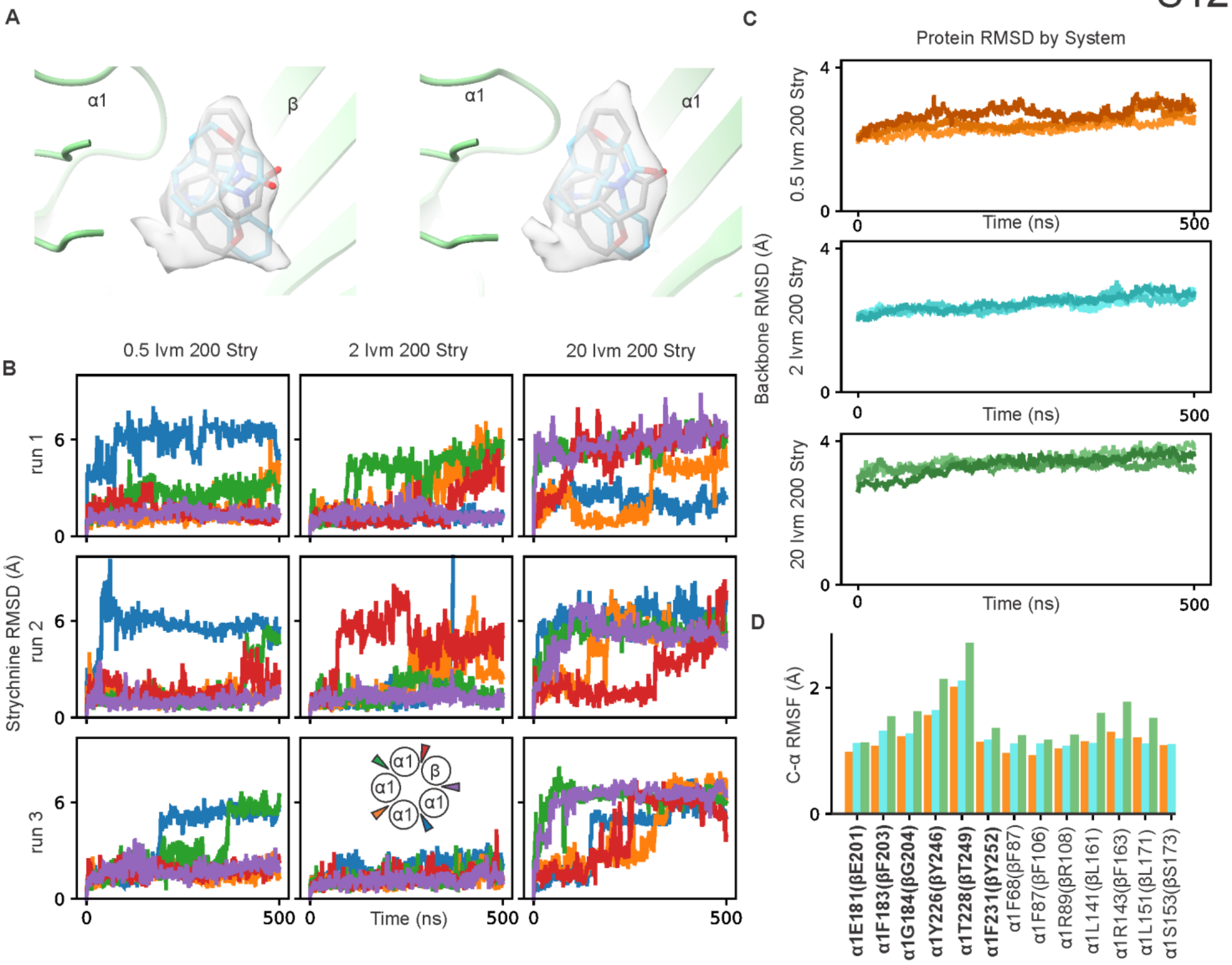

Supplementary Figure 12: Additional details of MD simulations.
Additional details of the simulation described in Fig. 5. A) Comparison of the vertical pose of strychnine and alternate horizontal pose of strychnine at the α1/β interface and the α1/α1 interface colored green in the figure legend of B). The cryo-EM density at these positions indicated heterogeneous strychnine binding. Though the final models had strychnine in the vertical position, we decided to test the possibility of a horizontal orientation at these interfaces in the simulations of GlyRα1β-20Ivm200Stry. However, this position was quite unstable as is seen in the purple and green lines of the GlyRα1β-20Ivm200Stry strychnine RMSD plots. B) The strychnine RMSD during the simulation is shown for all three simulations of each ligand condition. The colors correspond to the strychnine positions in the cartoon legend. C) The RMSD of the protein backbone for the three simulations in each condition. The different shades correspond to different simulation runs. D) The RMSF of selected residues near the binding pocket. Primary interface residues are bolded. Residues in Loop C and Loop E are more mobile, suggesting these elements are changing over the course of the simulation.

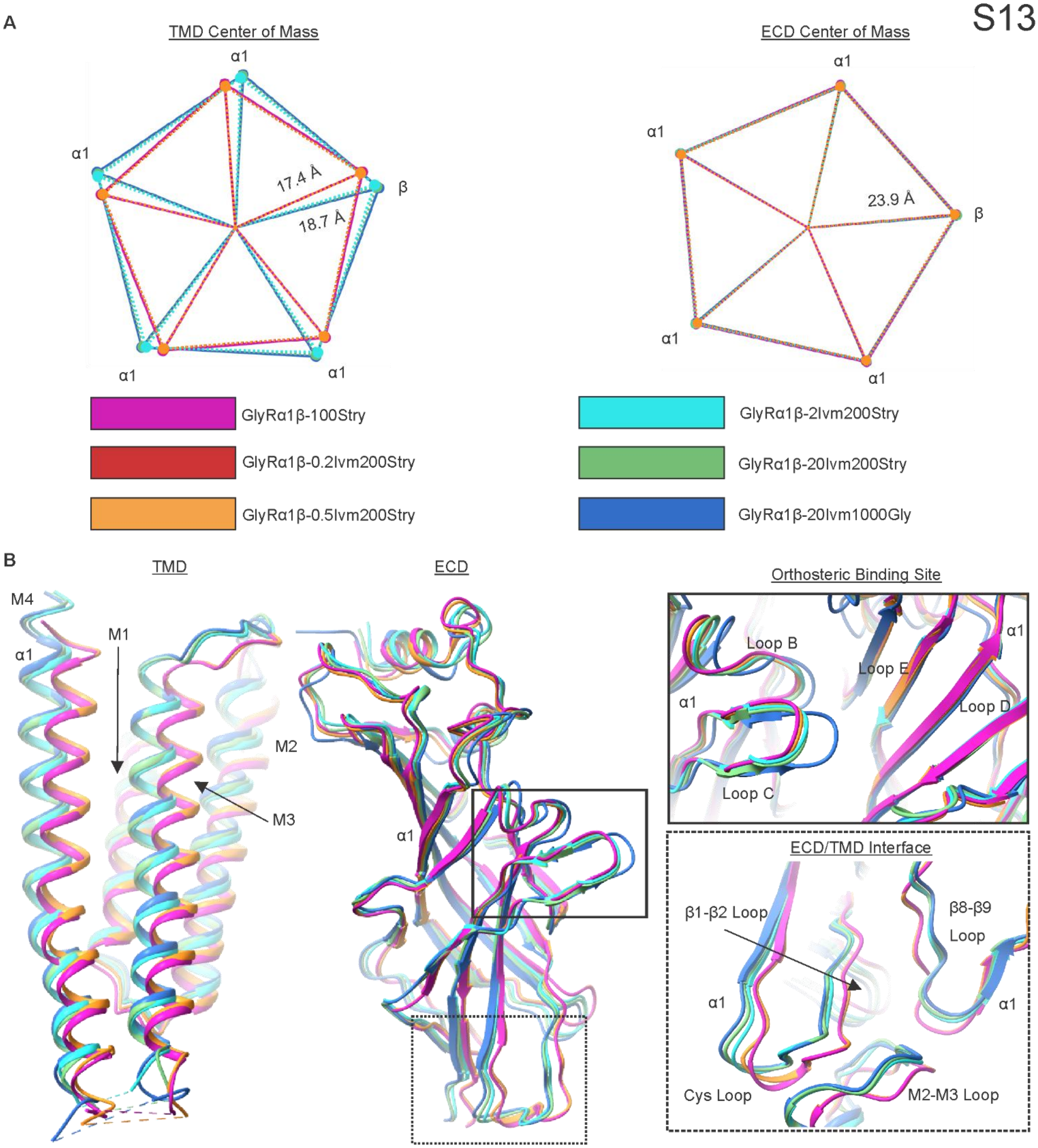

Supplementary Figure 13: Additional details of allosteric movements.
Description of the allosteric movements between different ligand states. Alignments were done using full pentamers even when only one subunit is shown. A) The COM for each subunit is shown for C-α atoms within the TMD (left) and ECD (right). All the states are shown, though they are tightly overlaid in the figure. Within the TMD, the COM of each subunit moves symmetrically outward and clockwise between the closed state and desensitized state. There is no change in the COM of the ECD between states. Both the TMD and ECD also undergo rigid rotations about the COM that vary between states as described in the text. Note, as there are no external references, one cannot distinguish the absolute rotation of the TMD or ECD. B) Differences between alignments within the TMD, ECD, orthosteric binding site, and the ECD/TMD interface. GlyRα1β-0.5Ivm200Stry is similar to GlyRα1β-100Stry throughout the protein. GlyRα1β-2Ivm200Stry and GlyRα1β-20Ivm200Stry resemble GlyRα1β-20Ivm1000Gly within the TMD, GlyRα1β-100Stry at the binding pocket, and is intermediate to the two at the ECD/TMD interface. GlyRα1β-0.2Ivm200Stry and GlyRα1β-20Ivm200Stry are not shown.

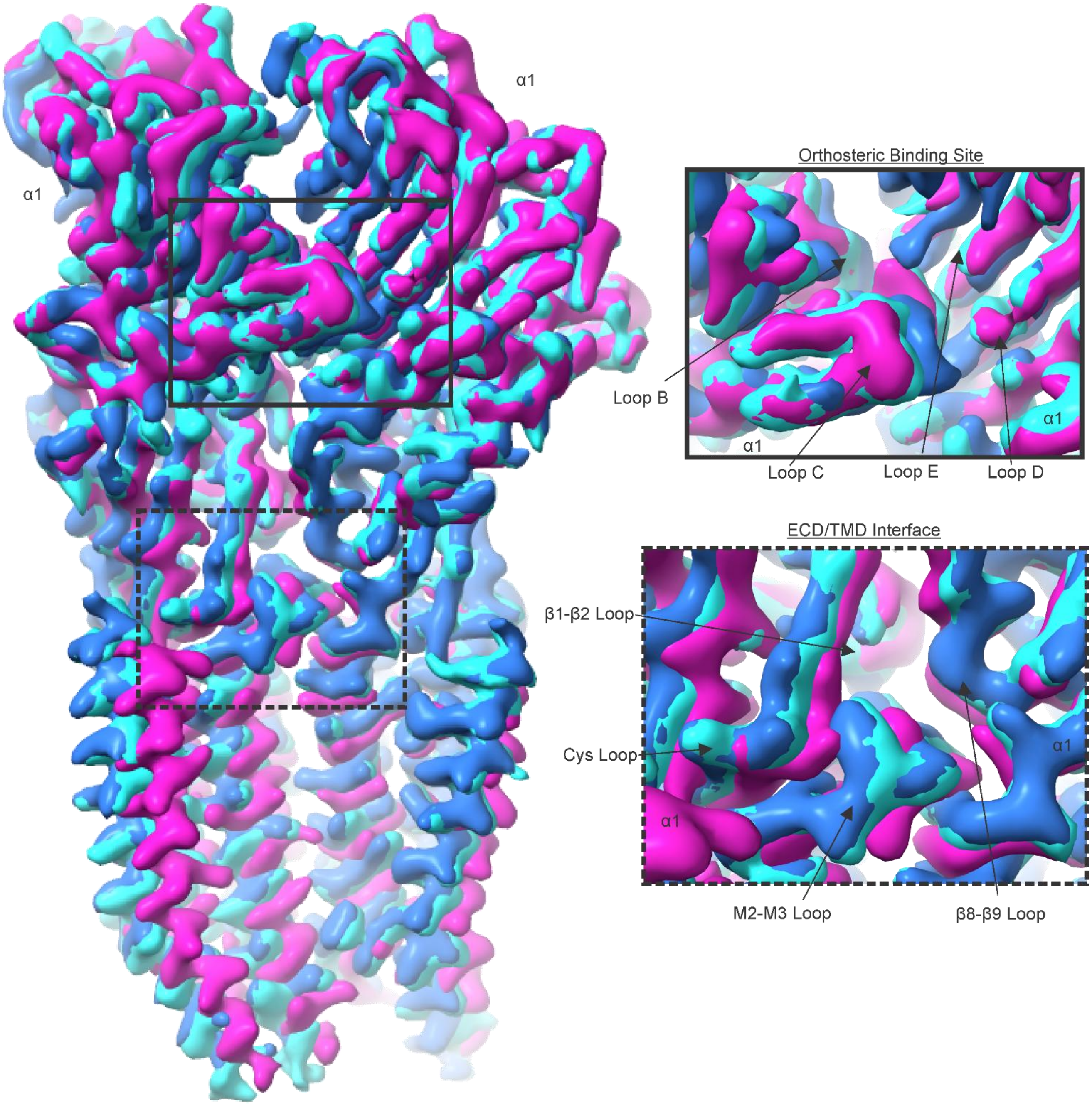

Supplementary Figure 14: Comparison of map density at an α1/α1 interface across functional states. GlyRα1β-100Stry, GlyRα1β-2Ivm200Str, and GlyRα1β-20Ivm1000Gly, were aligned across the entire pentamer and the corresponding maps show differences in subdomains of the protein. GlyRα1β-2Ivm200Stry aligns best with GlyRα1β-100Stry through most of the ECD, is intermediary at the ECD/TMD interface, and aligns best with GlyRα1β-20Ivm1000Gly in the TMD.

	1.5 minute	Imax
t-statistic	-2.672	-2.448
Degrees of Freedom	8	8
p-value	0.02826	0.04
95% Confidence Interval	[-2.49, -0.18]	[-3.26, -0.09]

Supplementary Table 1: Significance statistics for Fig. 1C.

Sample	GlyRα1β-0.2Ivm200Stry	GlyRα1β-0.5Ivm200Stry	GlyRα1β-2Ivm200Stry	GlyRα1β-20Ivm200Stry
PDB id	9PKW	9PKX	9PKY	9PKZ
EMDB id	71705	71706	71707	71708
Data collection and processing				
Microscope and location	Titan Krios (CWRU)	Titan Krios (CWRU)	Titan Krios (CWRU)	Titan Krios (NYSBC)
Magnification	105000	105000	10500	105000
Voltage	300	300	300	300
Data collection mode	counted	counted	counted	counted
Camera	K3	K3	K3	K3
Physical pixel size	0.84 Å/pixel	0.84 Å/pixel	0.84 Å/pixel	0.82 Å/pixel
Defocus range (uM)	−0.8 to −1.6	−0.8 to −1.6	−0.8 to −1.6	−0.8 to −1.6
Number of movies	8,740	9,711	25,020	38,701
Micrographs with reasonable CTF FOM	7,944	8,328	20,921	27,561
Dose per frame	1.2 $e^-/Å^2$	1.2 $e^-/Å^2$	1.2 $e^-/Å^2$	1.2 $e^-/Å^2$
Number of frames/movie	50	50	50	50
Initial particle number	2,478,973	1,804,275	5,686,816	4,775,310
Final particle number	73,223	94,460	93,904	129,399
Symmetry	C1	C1	C1	C1
Resolution (unmasked, Å)	3.32 Å	3.42 Å	3.32 Å	3.37 Å
Resolution (masked, Å)	2.86 Å	2.86 Å	2.89 Å	2.89 Å
Refinement				
Initial model used	GlyRα1β-100Stry	GlyRα1β-100Stry	GlyRα1β-20Ivm1000Gly	GlyRα1β-20Ivm200Gly
Composition	4α1:1β	4α1:1β	4α1:1β	4α1:1β
Protein residues	1677	1683	1698	1714
Non Hydrogen atoms	13791	13843	14301	14429
Glycan (NAG) (molecule)	5	5	6	5
Strychnine (molecule)	5	5	5	5
Ivermectin (molecule)	0	0	5	5
Bonds (RMSD)				
Length (Å) (# > 4σ)	2	0	0	0
Angles (°) (# > 4σ)	1	3	3	2
Ramachandran plot (%)				
Outliers	0	0	0	0
Allowed	1.75	1.38	2.15	2.54
Favored	98.25	98.62	97.85	97.46
Rotamer outliers (%)	0	0	0	0
Molprobity score	1.11	1.27	1.26	1.42
Molprobity clashscore	3.19	5.05	4.53	5.25

Supplementary Table 2: Cryo-EM data collection, refinement, and validation statistics.

Welch’s t-test t-statistic	3.62
p-value	.0167
Degrees of freedom	4.74
n	5
Effect size (Cohen’s d)	2.29
95% confidence interval for mean difference	[152.99, 947.41]

Supplementary Table 3: Significance statistics for Fig. 5B.

Interface 1	Interface 2	Tukey's HSD pairwise statistic	p-value	Confidence Interval	Effect size (Cohen's d)
GlyRα1β-0.5Ivm200Stry (α1/α1)	GlyRα1β-20Ivm200Stry (α1/α1)	0.641	0.029	[0.041, 1.242]	1.74
GlyRα1β-0.5Ivm200Stry (α1/α1)	GlyRα1β-20Ivm200Stry (β/α1)	1.636	0.000	[0.786, 2.485]	4.53
GlyRα1β-0.5Ivm200Stry (β /α1)	GlyRα1β-20Ivm200Stry (β/α1)	1.265	0.008	[0.224, 2.305]	3.04
GlyRα1β-0.5Ivm200Stry (α1/β)	GlyRα1β-20Ivm200Stry (β/α1)	1.808	0.000	[0.767, 2.849]	7.78
GlyRα1β-2Ivm200Stry (α1/α1)	GlyRα1β-20Ivm200Stry (β/α1)	1.431	0.000	[0.581, 2.281]	3.37
GlyRα1β-2Ivm200Stry (β /α1)	GlyRα1β-20Ivm200Stry (β/α1)	1.744	0.000	[0.704, 2.785]	11.67
GlyRα1β-2Ivm200Stry (α1/β)	GlyRα1β-20Ivm200Stry (β/α1)	1.831	0.000	[0.790, 2.871]	5.38
GlyRα1β-20Ivm200Stry (α1/α1)	GlyRα1β-20Ivm200Stry (β/α1)	0.994	0.012	[0.144, 1.844]	3.23
GlyRα1β-20Ivm200Stry (α1/β)	GlyRα1β-20Ivm200Stry (β/α1)	1.2	0.014	[0.160, 2.241]	8.03

Supplementary Table 4: Significance statistics for Fig. 5C. There were 9 samples for each (α1/α1) interface and 3 samples of the (β/α1) and (α1/β) interfaces and 36 degrees of freedom.
